# Improving resilience of Arabica coffee plantations by cultivating F1 hybrid varieties: challenges to future seed production and enhancing farmers’ access to modern varieties

**DOI:** 10.3389/fpls.2026.1905477

**Published:** 2026-09-02

**Authors:** Frédéric Georget, Hervé Etienne, Edgardo Alpizar, Philippe Courtel, Ana Laura Sandoval, Jefferson Guato, Mariana Tovar, Gonzalo Contreras, Juan Carlos Herrera

**Affiliations:** 1 CIRAD, UMR DIADE, Montpellier, France; 2 DIADE, Univ Montpellier, CIRAD, IRD, Montpellier, France; 3 ECOM, Plant material, Exportadora Atlantic, Managua, Nicaragua; 4 Nestlé, Agricultural Operations, Mexico City, Mexico; 5 Nestlé, R&D Experimental farms, Quebedo, Ecuador; 6 Nestlé, Coffee & Cocoa Program, Mexico City, Mexico; 7 Nestlé R&D, Plant Science Center – NIAS, Tours, France

**Keywords:** adaptability, agroforestry, clonal propagation, coffee sustainability, hybrid seed gardens, hybrid varieties, male sterility

## Abstract

The production of F1 hybrid Arabica varieties is a key strategy to improve the resilience, competitivity and sustainability of coffee cultivation. Indeed, under both full sun and agroforestry systems, these varieties have performed better than traditional varieties in terms of higher yields, cup quality, and enhanced tolerance to biotic and abiotic stresses, including remarkable adaptation to the shady conditions of agroforestry. Until recently, somatic embryogenesis was a key cloning methodology that made it possible to start propagating the first selected F1 hybrid. However, this *in vitro* method of multiplication is complex and costly, technically demanding and hence limited to a few high-tech micropropagation laboratories. Horticultural vegetative propagation techniques such as rooted mini-cuttings have been developed to reduce the cost. They provided a scalable solution for larger-scale dissemination by spreading it across numerous regional nurseries at less cost but still too high, thereby contributing to the popularization and adoption of F1 hybrids. More recently, a major innovation for the global coffee sector was the arrival of new Arabica F1 hybrids with similar agronomic performances but produced by seed and using male sterility. The cost of seed propagation is low, similar to the production of seedlings of conventional pure lines plus seeds are easy to disseminate, while clonal methods ensure total genetic uniformity so, depending on the production goals, local constraints and the target market, the two approaches may be complementary. This methodological paper focuses specifically on F1 hybrid Arabica coffee seed production. Changes to breeding strategies and the different propagation techniques used to develop and disseminate F1 hybrid varieties are described, together with the advantages and limitations, with emphasis on biotechnological approaches, horticultural clonal propagation methods and recent advances in male sterility systems. Technical aspects aimed at improving the use of seed gardens and optimizing hybrid seed production in coffee are reviewed. F1 hybrid seed technology represents a promising development for coffee breeding and propagation, allowing access to high-performing and more resilient cultivars, hence supporting farmer’s transition toward climate-smart and sustainable coffee production systems.

## 1 Introduction

Climate change is already having a dramatic impact on agriculture, reflecting the close link between climate (temperature and precipitation in particular) and productivity. These effects will likely have the greatest consequences in the least developed countries in tropical zones where productivity will decrease. Indeed, agricultural output in these tropical countries is expected to decline by 20% by 2080 due to climate change and yields could decrease by an average of 15% (Masters et al., 2010).

Green bean yield in coffee is tightly linked to climatic variability and is thus strongly influenced by natural climatic oscillations. Among the two commercial coffee species, C. arabica and C. canephora, the Arabica species is the most sensitive in terms of disease tolerance and the least resilient. The impact of climate change will be negative, i.e. reduction of suitable areas for cultivation, reduction of farmers’ income and well-being, increasing damage by pests and/or diseases, but also positive, i.e. more suitable new production areas at high altitudes, or the predicted favorable effect of high CO2 concentrations on productivity. Even if the effects of climate change on the cultivation of the Arabica coffees have been widely reviewed by different authors (Davis et al., 2012; Ovalle-Rivera et al., 2015; Bilen et al., 2023), predicting future trends is complex since the relationships between climatic parameters and farming systems are further complicated because these environmental factors influence plant development in different ways during the growth stages of the coffee crop (Camargo, 2010).

Given the adverse effects of climate change, there is a global consensus that more exposed coffee cultivation regions may require specific policies and strategies to ensure that expansion of future coffee cultivation takes place in climatically, pedologically and ecologically suitable areas. Climate challenges are pushing countries to adopt more resilient, high-performing genetic material. In this context, developing F1 coffee varieties that can be developed quickly in response to changing conditions and are specifically designed for agroforestry production is becoming a priority for plant breeders (Ovalle-Rivera et al., 2015).

In the last 20 years, F1 Arabica coffee hybrids have produced higher yields, become more resilient to climate changes and their cup quality proved to be better than that of conventional cultivars (Bertrand et al., 2006; Van der Vossen et al., 2015). The same hybrids also showed considerable promise in terms of the economic and environmental sustainability of the farms that adopted them, thanks to their better resilience and adaptability to agroforestry systems compared to the traditional pure-line cultivars (Bertrand et al., 2011; Meter et al., 2023). The improved stress resilience of F1 Arabica hybrids is due to their hybrid vigor. However, few authors have attempted to understand the genetic and physiological mechanisms underlying heterosis. Some authors suggest it is related to the differential regulation mechanisms associated with carbon metabolism, photosynthetic efficiency and the circadian clock (Toniutti et al., 2017; Gamboa-Becerra et al., 2021).

To date, due to their heterozygous structure, large-scale production of F1 hybrid coffee plants has relied exclusively on clonal propagation, either using costly *in vitro* techniques, i.e. micro cuttings, somatic embryogenesis, or horticultural propagation systems, i.e. rooted cuttings, or grafting, in greenhouses that require dedicated infrastructure and specialized know-how. Although these approaches currently enable the production of several million plants per year, they remain insufficient to support widespread adoption by producers.

In particular, smallholder farmers often have limited access to credit, making the investment required for plantation renewal with F1 hybrid material economically prohibitive compared to the use of pure-line varieties that can be readily propagated by seed on the farm. Consequently, the major challenge associated with the deployment of Arabica coffee F1 hybrids is developing efficient and economically viable large-scale clonal propagation systems capable of competing with conventional seed-based propagation methods.

However, the situation will be quite different if F1 coffee hybrids can be produced by seed. The discovery of a male sterility system in coffee means a faster and cheaper way of multiplying F1 Arabica hybrids is now available. This improvement opens the way to reproduce F1 hybrids in a natural way offers huge advantages as it will be possible to produce large quantities using seeds that are easy to handle, store, and transport, and much more affordable for farmers (Georget et al., 2015b and 2019). Despite these advantages, the use of male sterility for efficient production of F1 hybrid seeds in coffee remains largely unexploited.

The present article is intended as a narrative review based on the authors’ expertise in coffee breeding and propagation, combined with an analysis of the peer-reviewed literature and selected technical reports relevant to Arabica F1 hybrid seed production. Rather than providing a systematic review of the literature, our objective is to summarize the key scientific and technical advances that determine the successful production and dissemination of F1 hybrid coffee varieties.

Specifically, we first provide an overview of the evolution of coffee breeding strategies, the emergence of the new generation of F1 hybrid varieties, and their contribution to more sustainable coffee production systems. Second, we present the main technical aspects involved in the establishment and management of coffee seed gardens and the optimization of high-quality hybrid seed production. Finally, we discuss strategies to improve the large-scale deployment of F1 cultivars and to facilitate their adoption by farmers in a context where climate resilience, sustainability, and innovation are becoming increasingly important.

## 2 Overview of F1 hybrid development in Arabica coffee

### 2.1 Changes in Arabica coffee breeding strategies

Between the late 17^th^ and the 20^th^ century, coffee cultivation spread across the global tropics from two tall cultivars (Typica and Bourbon) that originated from Yemen and were selected by empirical mass selection under full sun conditions. The first varietal revolution took place between 1970s and 1980s and was based on dwarf selections (Caturra cv.), that first developed in Brazil from mutation of Bourbon and Typica cultivars. The exploitation of the Caturra dwarf mutation led to the creation of dwarf cultivars derived from crosses between Caturra, Typica and Bourbon, i.e. Mundo Novo, Catuai, Caturra. In 1970, the Coffee Rust Research Centre of Brazil (CIFC) released a number of crosses between selected Timor hybrid genotypes (CIFC832/1, CIFC832/2 and CIFC1343) and the compact Caturra, Catuai and Villa Sarchi varieties. After more than a decade of pedigree selection, this release enabled coffee research centers in Latin America to develop compact coffee cultivars that are resistant to coffee leaf rust, Catimor and Sarchimor (Silva et al., 2006). The productivity and CLR-resistance of these new dwarf cultivars have enabled the intensification of coffee cultivation in Latin America, leading to increased productivity under full sun conditions but with the use of large quantities of chemical fertilizer, herbicide and fungicide. This period is known as the Green Revolution. The third stage in coffee production begun in 1990 with the development of Arabica coffee F1 hybrids derived from a cross between two pools of genetic material, dwarf Arabica cultivars and introgressed dwarf cultivars derived from the Timor hybrid, and wild coffee trees from Ethiopia or Sudan. F1 hybrids combine higher productivity, better adaptability to agroforestry, greater tolerance to pests and diseases, and better cup quality and are thus a promising alternative to conventional cultivars. This method of selection enables new varieties to adapt more quickly to global changes than traditional breeding, which in the past, took several decades. Today, modern breeding strategies have extended the selection of the new Arabica F1 hybrid varieties to ideotypes exhibiting not only high yield, good vigor and improved disease tolerance, but also high physiological resilience against climate change, i.e. heat and drought stress, through better adaptation to shaded systems (Van der Vossen, 2018; Breitler et al., 2022). Different breeding teams have already integrated genomic-based strategies, i.e. genomic and phenomic prediction, genome-wide association, genomic and genetic selection, to improve selection in coffee with the goal of saving time, reducing the number of breeding populations and finally increasing the cross efficiency of future coffee cultivars (Sant’Ana et al., 2018; Gimase et al., 2020; Mbebi et al., 2022; Da Silva et al., 2024; Heslot et al., 2015).

### 2.2 Agronomic performance of Arabica F1 hybrids

The first agronomic studies on Arabica coffee F1 hybrid varieties were carried out twenty years ago in Costa Rica by the CIRAD, CATIE and PROMECAFE research network with the characterization of hundreds of F1 progenies generated from crosses between Ethiopian wild accessions and traditional or improved American lines derived from the Timor hybrid (Bertrand et al., 2005). The same study showed that the selected hybrids produced from 11% to 47% more coffee than the parental control lines but also exhibited high fertility and bean quality. The heterozygosity displayed by the hybrids was linked with the production of longer primary branches with more productive nodes. Subsequent studies confirmed the homeostatic nature of the F1 hybrids under different altitudinal ranges when compared to the traditional varieties. Their organoleptic evaluation showed similar sensory specificities between Arabica F1 hybrids and the best traditional cultivars, and some hybrids exhibited better cup quality (Bertrand et al., 2006). The resilience of Arabica F1 hybrids has been studied in agroforestry systems (AFS) where hybrids proved to be well adapted. F1 hybrids yield significantly more in full sun (+34%) or shaded conditions (+58%) compared to the traditional American cultivars (Bertrand et al., 2011). Further studies suggested that physiological adaptation mechanisms combined with the circadian clock in coffee F1 hybrids could explain why they performed better than inbred lines under stressful conditions (Toniutti et al., 2017; 2019). Bertrand et al. (2021) showed that F1 hybrids between selected Catimor lines and Ethiopian genitors produced by CENICAFE in Colombia were more productive under both shade and full sun than their parental lines, suggesting that selection under shade could be used to develop varieties better adapted to AFS. The better adaptation of hybrids to AFS reflected by the ability to maintain higher yield under shade than the American cultivated pure lines varieties may be linked to the origin of the Ethiopian wild accessions used as genitors in mesophilous forests (Sylvain, 1955). Recent studies in Vietnam showed that some F1 hybrids are tolerant to drought stress while maintaining high yields in AFS (Sarzynski et al., 2024).

The particular agronomic performances of Arabica F1 hybrid produced by clonal propagation mentioned above are also shared by Arabica F1 hybrids produced by seed using male sterility. For example, Starmaya^®^ F1 hybrid, created by CIRAD in collaboration with ECOM group in 2019, showed superior agronomic characteristics in terms of vigor, bean size and yield than the Marsellesa^®^ cultivar used as a parent or the Caturra cultivar (Marie et al., 2020). Starmaya^®^ also has a better cup quality than other traditional cultivars.

Given the importance and potential of F1 hybrids as a new generation of coffee varieties, they may contribute to the development of more climate-resilient production systems and represent a promising component of future climate-smart coffee strategies aimed at reducing the impacts of ongoing climate change. Next, we give an overview of the different methods currently used to propagate coffee F1 hybrids, with their respective advantages and limitations in a context where coffee renovation areas obviously lack new resilient planting material.

## 3 Propagation methods for Arabica F1 hybrids

**Figure 1:**
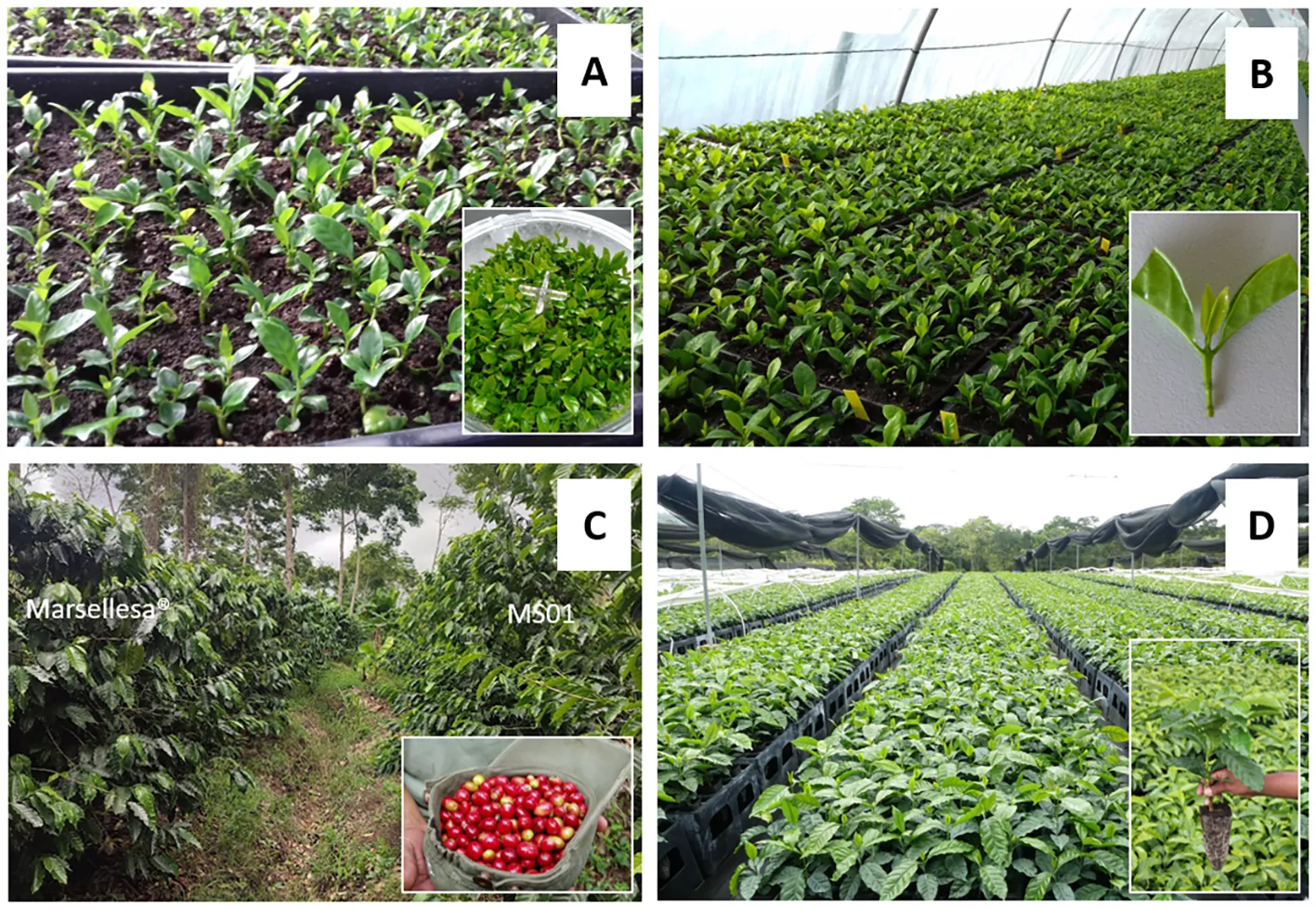
Different methods of propagation of Arabica F1 hybrids. **(A)** Direct sowing of somatic embryos in horticultural trays for germination **(B)** Horticultural propagation using the rooted mini-cuttings technique **(C)** Seed gardens using male sterility to produce F1 Starmaya^®^ seeds (Marsellesa^®^ x CIR-MS01) **(D)** Acclimation and hardening of six-month-old F1 Arabica plants in a sophisticated nursery using peat moss substrate and recyclable plastic tubes (Nicaragua).

Once an F1 hybrid variety was created, the only way to multiply it on a large scale was using a vegetative propagation method like somatic embryogenesis, or horticultural cloning like rooted cuttings or grafting. This was the case before the discovery of a male sterility system that offered new opportunities to multiply Arabica F1 hybrids in a natural way through seeds. We now describe alternative methods used for the multiplication of Arabica F1 hybrids (**Figure 1**).

### 3.1 Somatic embryogenesis

Somatic embryogenesis (SE) was the main method used to propagate the first generation of Arabica F1 hybrids (**Figure 1A**). The coffee tree is one of the very few crops for which commercial application of this technology has been successful. SE ensures clonal fidelity and large-scale production of elite hybrid lines from leaf derived-embryogenic tissues (Menéndez-Yuffá et al., 2010). Today, this technique is relatively well controlled at industrial scale on *C. arabica* species by the CIRAD-ECOM public-private alliance in Montpellier (France) and Sebacco (Nicaragua) (Etienne et al., 2012, 2013, 2018, 2025; Bobadilla-Landey et al., 2013), and by NESTLE in Tours (France) on C. *canephora* species (Ducos et al., 2003, 2007; Etienne et al., 2018). However, because this technique still requires highly specialized laboratory facilities, trained personnel, and strict quality control, SE-derived plants remain expensive to produce and access to the technology is still limited to a few micropropagation platforms across the world.

Given these constraints to the SE process in both tissue culture laboratories and in the acclimatization process in the nursery, the sales price of a 6-month-old somatic seedling is still too high compared to the sales price of a pure line seedling cultivar (US$ 0.80–1 vs US$ 0.20-0.30) (Georget et al., 2017). A similar situation can be observed in many other crop species, including conifers, resulting in very high production costs for somatic seedlings (plants derived from SE) and hence an obstacle to the commercial exploitation of SE seedlings in general (Lelu-Walter et al., 2013).

### 3.2 Horticultural propagation

To reduce production costs and promote dissemination worldwide, a horticultural vegetative propagation technique complementary to SE was developed in which the young somatic seedlings produced in the tissue culture lab are used as an initial pool to be multiplied through rooted mini-cuttings (**Figure 1B**) (Georget et al., 2017). The combination of the two techniques enabled a considerably reduction in the sales price of a 6-month-old F1 Arabica plants derived from SE from US$ 0.8-1.0 to US$ 0.50-0.60, but still twice the price of a pure line seedling. Today, between two and three million superior Arabica F1 hybrids are produced using this method every year by ECOM-CIRAD alliance in different countries in Central America (Etienne et al., 2025).

Rooted mini-cuttings offer greater sales opportunities for F1 hybrid produced clonally but their relatively high cost continues to hinder their accessibility and further adoption by farmers. Indeed, small farmers do not systematically have access to bank loans and the cost of orchard renovation with Arabica F1 hybrid is still prohibitive compared with pure line varieties they can reproduce by seed themselves. The current production capacity is also insufficient with respect to the market demand for elite *C. arabica* material, which is estimated at 100 million F1 hybrid plants per year worldwide, i.e. 100 million ha of coffee plantations, 5% renovation per year (20-year life cycle) and the target of 10% with F1 hybrids (3,500 plants/ha). Therefore, the current challenge associated with the use of Arabica F1 hybrids remains the development of an efficient and profitable methodology for clonal mass propagation that can compete economically with traditional pure line seedlings.

### 3.3 Seed-based propagation using male sterility

In 2001, a natural Ethiopian mutant, called CIR-MS01, of *C. arabica* without any pollen production was found by CIRAD in a CATIE coffee field collection in Costa Rica. This individual was used by CIRAD breeders as a mother parent line to produce F1 hybrid seeds through the implementation of seed gardens (Georget et al., 2015b). Several years of field evaluation confirmed that the CIR-MS01 phenotype identified by CIRAD displayed complete and stable recessive sterility, whatever the environmental conditions and the age of the plant, juvenile or mature (Georget et al., 2019).

The molecular basis of the CIR-MS01 mutation is still not known, but preliminary microscopic and field analyses suggested that male sterility in this specific mutant results from a nuclear recessive mutation that induces a malfunction of the tapetum in the early microspore stages (Dufour et al., 1997). Recent collaboration between CIRAD and Nestlé research teams revealed that a single nucleotide polymorphism (SNP) located on chromosome 13, fully co-segregated with the male sterility trait in the CIR-MS01 plant (Mieulet et al., 2025).

An original method for Arabica F1 hybrid seed production, based on a seed garden established with two genitors including the MS as mother, was first described by Georget et al. (2015) and later applied on a large scale to produce the first commercial seeds of the variety called Starmaya^®^ (**Figures 1C, D**) (Georget et al., 2019, https://varieties.worldcoffeeresearch.org/). This new hybrid variety has attracted considerable interest within the coffee sector and represents a promising cultivar for the wider deployment of Arabica F1 hybrids among farmers worldwide (https://dailycoffeenews.com/2017/03/13/new-starmaya-hybrid-could-reshape-the-industry-says-world-coffee-research/).

### 3.4 Advantages and limitations of propagation using clonal methods versus seeds for large-scale dissemination of coffee F1 hybrids

There is no perfect way to propagate F1 hybrids. Whatever the propagation method chosen, it offers several advantages but also limitations as summarized in **Table 1**. The potential of production of F1 hybrid produced by clonal propagation is very limited due to the many complex, tedious and costly steps involved in their vegetative production through SE process. Today, only a few micropropagation laboratories worldwide are able to commercially produce such Arabica F1 hybrid and none of them is able to produce more than 3 million plants per year, which is a key constraint for scaling up and hence for their democratization. In terms of logistics and shipment, tissue culture labs and associated greenhouses and nurseries are generally located near large cities and airports, are easily accessible by road and have access to electricity, clean water and trained staff. The possibility to ship *in vitro* plantlets is uncertain due to the risk of material deterioration and loss during transport by air. In the case of ex-vitro plants, the cost of shipment is prohibitive due to the required high density at planting (around 3500 F1 hybrid plants/ha) and the resulting huge volumes of plants.

**Table 1:** Advantages and limitations of Arabica F1 hybrid clone vs hybrid seed.

Type	Advantages	Limitations
F1 hybrid clone	In F1 hybrids, clones are the master of class (both parents are selected by the breeder); Genetic uniformity: As clones, they are identical to the mother plant thus guaranteeing homogeneity in terms of yield and quality; Plant health & sanitary risks: As clones are produced in sterile conditions, they are generally pathogen free thus facilitating customs procedures; Very good cup quality (> 85 SCA quality score, Special coffee).	Not easy to propagate: SE is a technically complex technique (requires scientific knowledge and well-established protocols, a tissue culture lab, and sophisticated nurseries); Not easy to transport around the world (vitroplants are fragile, there is a risk of contamination, boxes holding containers and nutrient media are heavy); The production potential is limited: e.g. 2 to 3 million plants/year; High production costs: In Central America, the sales price is between US$ 0.5-0.6/plant (combining SE and rooted mini-cutting techniques); Risk of somaclonal variation: If SE is not mastered at industrial scale.
F1 hybrid seed	Easy to propagate: A 1-ha seed garden = 500kg of F1 seeds = Potential for 200 ha of renovated coffee orchards; Easy to handle, store and transport (in theory): After being harvested, F1 seeds can be processed then stored for more than 1 year in a controlled storage room; Low cost: The sales price per kilo of F1 seed ranges between US$50 and 100 (vs US$10 to 20/kg for traditional certified pure line seeds) and it takes about 2 kilos of seed to plant 1 hectare of coffee (~3,500 plants/ha); Good cup quality (80^+^ SCA quality score, HSB coffee)	The breeder can only choose the pollen donor: the mother parent is imposed by the male sterile mutants that were found in the international collection or in the wild but are very rare; Male sterility is still complex & mastering it to produce commercial F1 hybrid seeds is time consuming: an efficient pollination control system remains to be established; Production potential is still limited (e.g. 3 tons of seeds were produced in the framework of the CIRAD-ECOM collaboration project in 2025); Seed propagation can result in some heterogeneity among the progeny linked to cross-pollination events & residual heterozygosity of the MS Ethiopian mutant used as mother; Sanitary risks: Seeds may carry fungal, bacterial or viral diseases that can have negative phytosanitary impacts on the potential dissemination of seeds around the world.

Seed production using male sterility has the potential to facilitate wider distribution of F1 hybrids at low prices possible as it requires no costly infrastructure. Seeds can be stored for several months in cold-room and easily shipped all over the world. However, F1 hybrid seed dissemination may also be constrained by phytosanitary issues, as seeds can act as carriers of fungal, bacterial, or viral pathogens. In that case, appropriate seed health testing, certification procedures, and quarantine measures are therefore essential to ensure the safe international exchange and large-scale dissemination of this material.

Moreover, where sanitary regulations do not allow seeds to be imported, another alternative is to implement seed gardens directly in the different countries, so that F1 hybrid seeds can be produced locally with minor technology, as close as possible to coffee farmers. Based on current assumptions regarding annual demand for planting material (100 million trees), it is estimated that approximately 150 ha of seed gardens distributed across the main coffee-producing regions worldwide would be required to meet this demand, which is quite realistic in terms of both technical achievement and cost. Today, in the framework of collaboration between CIRAD and ECOM company, six hectares of seed gardens have been established in Nicaragua with a production capacity of around 3 tons of hybrid seeds per year (half ton of F1 seeds per ha), which would enable around 1200 hectares of new F1 coffee plantations to be re-planted every year (based on current nursery production estimates (approximately 1,500 seedlings per kilogram of seed) and a planting density of about 3,500 trees per hectare). Despite these promising results, F1 hybrid coffee seed production still faces substantial technical and agronomic challenges. Further research is required to optimize seed garden productivity through better flowering synchronization between parental genotypes, improved nutrient management, enhanced field management practices, and the development of efficient pruning strategies. Some technical aspects will be discussed later in this document in order to optimize the implementation of seed gardens production.

In terms of genetic conformity, if somatic embryogenesis is not well mastered at industrial scale, somaclonal variations may occur during the multiplication steps and lead to phenotypic variants (Etienne and Bertrand, 2001, 2003). However, it has been shown that somatic embryogenesis based on embryogenic cell suspensions cultured for less than six months with low auxin supply is efficient and reliable for true-to-type propagation of selected *C. arabica* F1 hybrids with over 99% of regenerated coffee trees in full conformity with the mother plant morphologically and with conservation of their original physiological and agronomic traits (Bodadilla-Landey et al., 2013 and 2015; Etienne et al., 2016 and 2018). Regarding Arabica F1 hybrid seed production using male sterility, some heterogeneity can also be observed in the progeny. In Starmaya^®^ for example, 2% to 5% of plants exhibit different phenotypes, e.g. tall plants, brown colour of leaf tips, sensitivity to rust, mainly due to cross potential events and residual heterozygosity of the two parents used for the cross “Marsellesa^®^ x CIR-MS01” (Georget et al., 2019).

### 3.5 The method of production depends of the target market

Given their nature and characteristics, both types of F1 hybrid plants (produced by clonal propagation or by seeds) would respond to different market expectations. For the “mainstream” coffee market, the objective is to produce conventional coffee at medium altitude, in large volumes and with standard cup quality that can be blended with other coffees if necessary, and sold at the market price. Both pure line and F1 hybrid seed varieties perfectly suit the commercial objectives and production requirements of this target market in terms of quality and volumes.

The cup quality of F1 hybrid produced by clonal propagation is generally much better than that of F1 hybrid seeds because both parents are selected by the breeder for this specific trait. This coffee will be grown under shade at high altitudes, and the coffee will be sold on the market with quality certifications, e.g. Rain Forest Alliance, Fairtrade, 4C, C.A.F.E. Practices, Nespresso AAA Sustainable Quality program, and as “specialty coffee” for a very high premium price that will help make the coffee farm more profitable and sustainable.

**Figure 2:**
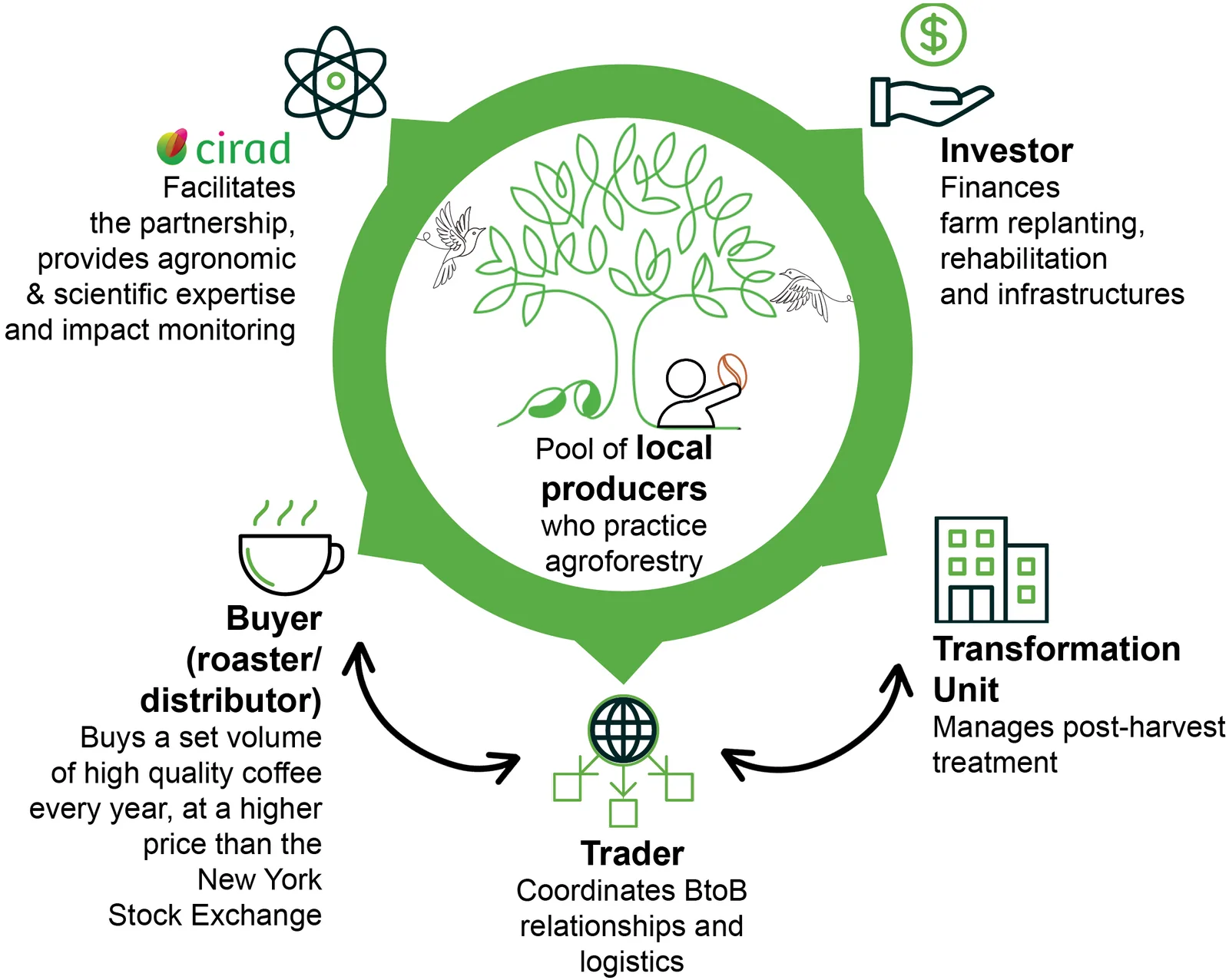
The new AFS business-driven cluster concept developed by CIRAD) A virtuous loop of stakeholders for fairer distribution of the added value throughout the coffee chain to increase coffee farmers’ income.

An innovative concept was also recently developed by CIRAD to provide high-quality coffees to consumers in Europe, USA and Asia (**Figure 2**). The concept, which is called “AgroForestry Systems (AFS) business driven cluster” is based on linking the producer to the final seller around a certified product, with high cup quality that results from sustainable agriculture based on the association of a *terroir*, agroforestry practices and the use of new coffee varieties that are more pest tolerant and more resilient to climate change (Meter et al., 2023). Both F1 hybrid produced clonally and by seeds perfectly match the commercial objectives and production requirements of the AFS business-driven cluster. The benefits and opportunities of AFS practices in coffee are reviewed and summarized by Lambot et al. (2017) and Koutouleas et al. (2022).

To illustrate this innovative concept, a 1,350-ha prototype was set up in Nicaragua in 2018, first using the pure-line variety Marsellesa^®^, to which the F1 hybrid Starmaya^®^ was added in 2022 to produce an exceptional coffee marketed by the industry (Nespresso’s Master Origin Nicaragua capsules). The sale of the coffee produced by the Agroforestry coffee cluster to Nespresso has introduced a ‘business driven’ dimension to the project i.e. better distribution of added value throughout the value chain by paying farmers more for green coffee (Meter et al., 2023). This model is intended to be replicated in new *terroirs* through projects covering approximately 1,000 to 2,000 ha, targeting a minimum annual production of 2,500 tons of green coffee, the threshold required to market a distinct “*cru”* (Bertrand et al., 2019).

As explained by Meter et al. (2023), the concept has the potential to be transferred and implemented in other coffee-producing countries, although its successful adoption will depend on local agroecological, economic, institutional and regulatory conditions. Smallholder farmers are not excluded from this approach, as they can be integrated into the cluster provided, they cultivate the same hybrid variety, comply with a common set of production standards and are clustered in the same business unit. In this case they would benefit from significant investment for the renovation of their parcels, and from the good price of the resulting coffee.

Until now, the development of large agroforestry clusters based on F1 hybrid varieties has been hampered mainly by to the limited availability and the high cost of F1 hybrid plants which represents a huge investment for investors. This situation is changing with the emergence of F1 hybrids produced by seed, such as the variety Starmaya^®^ and Mariana^®^.

The establishment of coffee agroforestry business-driven cluster as a new production model based on the use of high performing and resilient F1 hybrid varieties is a response that is preferred by donors and stakeholders in the coffee industry to address both the challenges of the agroecological transition and ongoing climate change, while simultaneously promoting coffee quality and traceability. The original organization of the value-chain around big agroforestry clusters, orchestrated by key stakeholders (Producers, Investor, a Business unit, Trader and Buyer), guarantees significant added-value for all, leading to a virtuous loop. It carries the guarantee of high environmental standards, protecting biodiversity and mitigating the effects of climate change.

## 4 F1 hybrid seed production: current methodologies

### 4.1 Overview of the biological mechanisms and methods used for hybrid seed production

For both field and vegetable crops, the use of hybrids to improve plant vigor and productivity is a recognized strategy to exploit diversity for commercial purposes. Developing hybrid varieties implies crossing parental plants exhibiting contrasting levels of genetic diversity to achieve maximum hybrid vigor. Mastering the biological mechanism controlling self-pollination is the first step of the production process. For high quality hybrid seed production, it is also necessary to optimize the associated factors that determine the growth, flowering synchrony and reproductive compatibility of parental genotypes (Longin et al., 2012; Fu et al., 2015).

#### 4.1.1 Reproductive mechanisms for pollination control

From a biological context, plants have evolved to develop a varied set of mechanisms to favor - or avoid - cross pollination. Factors like floral biology, the mating system, or even agronomic conditions determine the way seeds should be produced in the best conditions.

**Figure 3:**
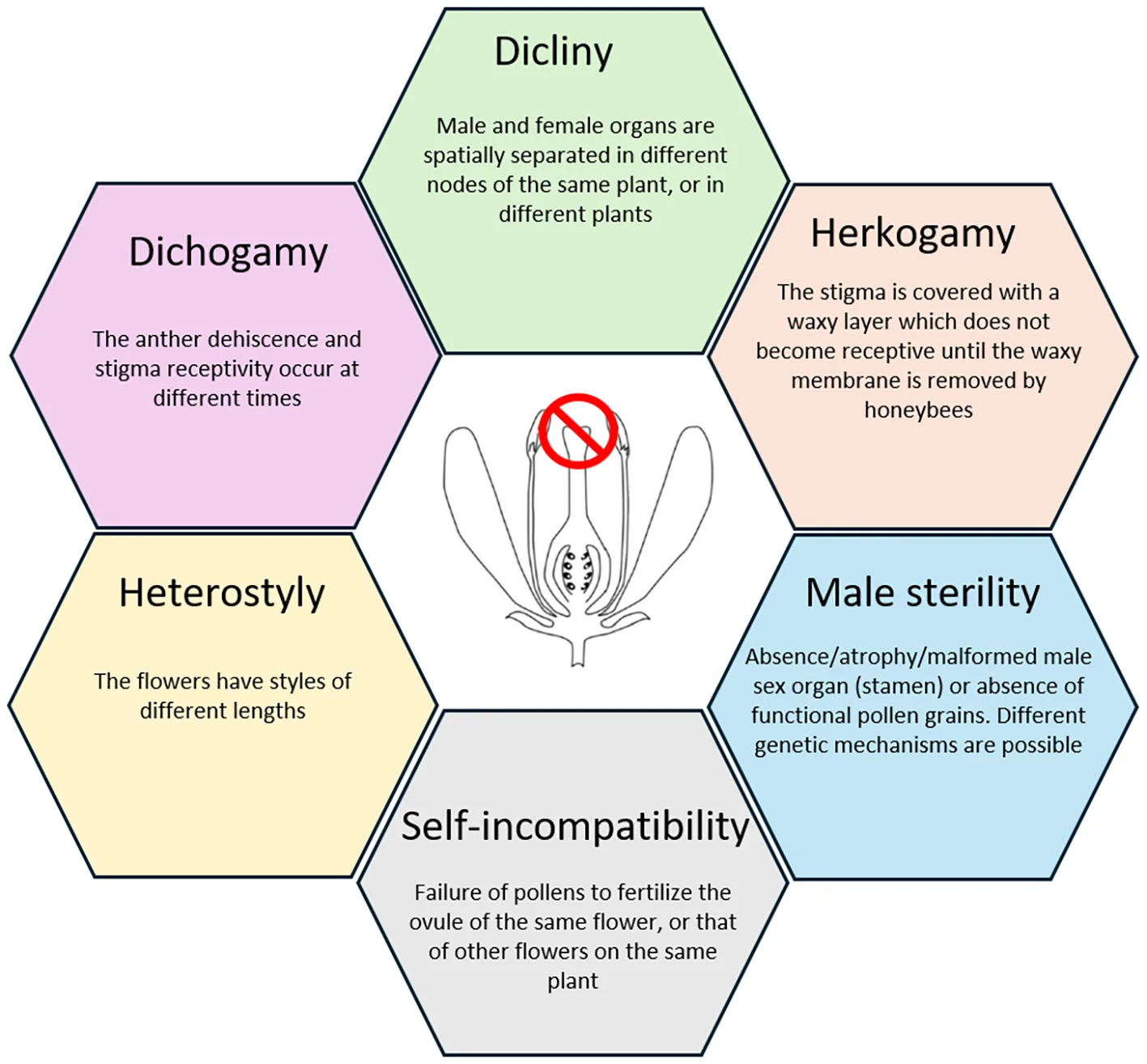
Description of different biological mechanisms that limit cross pollination in cultivated crops.

Among the biological mechanisms that control seed production by plants, some are related to floral structure, i.e. dicliny, herkogamy, male sterility or heterostyly, to the timing of development, i.e. dichogamy, or genetic incompatibility mechanisms, i.e. self-incompatibility, male sterility. These mechanisms are widely reviewed by Chakrabarty et al, (2023) and the main differences are summarized in **Figure 3**.

Hybrid varieties in vegetable crops have played a key role in increasing the efficiency and quality of vegetable production systems (Da Silva, 2024). Therefore, production of hybrid seeds in vegetables is a good illustration of the good practices required for a hybrid seed garden. Colombo and Galmarini (2017) compared the different systems used for pollination control in 26 of the main vegetable crops. Among the natural pollination control systems found in vegetable crops, cytoplasmic male sterility remains the most frequently applied for commercial purposes, i.e. in 14 out of 26 crops examined. Interestingly, spontaneous or induced genic male sterility is present in most species, i.e. in 24 out of 26 crops examined, but the system is applied commercially in only 4 out of 26 species, cabbage, leek, chicory and pepper.

Self-incompatibility is another well documented system that has been used for commercial seed production in 6 out of 26 species with good results in some popular crops like cabbage, broccoli, and radish (Murase et al., 2024). Pollination control by artificial systems, i.e. manual emasculation, chemical-hybridizing agents, offers an alternative when natural systems are not efficient or simply absent. Manual control remains the most frequently applied system for commercial purposes while chemical methods are available for only a few species due to the difficulties involved in implementing them in products for sale.

The main advantages and limitations of the current systems used to restrict the production of pollen in hybrid seed gardens of different crops are summarized in **Table 2**. Whatever the system, the duration of stigma receptivity and pollen availability are critical variables that determine the success of seed production. Indeed, stigma receptivity can be significantly affected by climatic conditions during flowering, and its duration varies considerably among species, from three days up to more than a week. Similarly, pollen vigor and viability are essential to accurately schedule hybridization. Pollen viability varies greatly among crop species (from a few minutes to several weeks) and can be influenced by climatic factors but also by the nutritional and disease status of the plant (see review by Frankel and Galun, 2012). Further, controlling pollen storage conditions facilitates hybrid seed production from one year to the next (Calic et al., 2021). Even if the flowering pattern differs in the two parental lines, it is always possible to improve the fertilization rate by manually or by allowing/promoting natural pollination by the wind and/or insects (Sundareswaran et al., 2023).

**Table 2:** Advantages and limitations of the biological and induced systems used to prevent the production of pollen by the female parent in a hybrid seed garden.

Biological system	Advantages	Disadvantages
With genetic determinism		
Genic male sterility	Some male sterility genes change their expression under different environmental condition making MS a reversible process. Genetic transformation for MS is available in different species.	The majority of the ms genes are recessive. A lack of suitable markers, as well as laborious maintenance processes and poor free outcrossing in certain highly self-pollinated species
Cytoplasmic male sterility	CMS can be obtained through wide crosses as a result of intra/interspecific or intergeneric nuclear/cytoplasmic incompatibilities. Genetic engineering has been successfully opening a vast rang of possibilities.	Hybrid seed production using CGMS is technically complex. The mechanism involved in CMS has not been elucidated. Some adverse effects of the sterile cytoplasm have been observed. Male sterile cytoplasms and restorer genes are not always available.
Self -incompatibility	Only two self-incompatible lines carrying different S alleles are necessary	Hybrid seed production requires the maintenance of self-incompatible lines. SI adoption for hybrid seed production requires high maintenance cost for SI lines, Expression of self-incompatibility would be affected by environmental and genetic factors.
Without genetic determinism		
Manual control	MC is well adapted for species that will produce many seeds from each pollination. Proven efficacy for hybrid seed production in both field and vegetable crops	Method is labor intensive and requires highly skillful human resources. Not suitable in species with small hermaphrodite flowers.
Chemical control	This technology is ease of making and evaluating hybrid combinations. It is labor efficient for seed production and no need for developing male sterile and restorer lines.	Can induce tissue toxicity effects on the female or F1 seed. Requires use of precise stage of plant development for working. High interaction with genotypes and environmental conditions

#### 4.1.2 Field methods used in seed production

**Figure 4:**
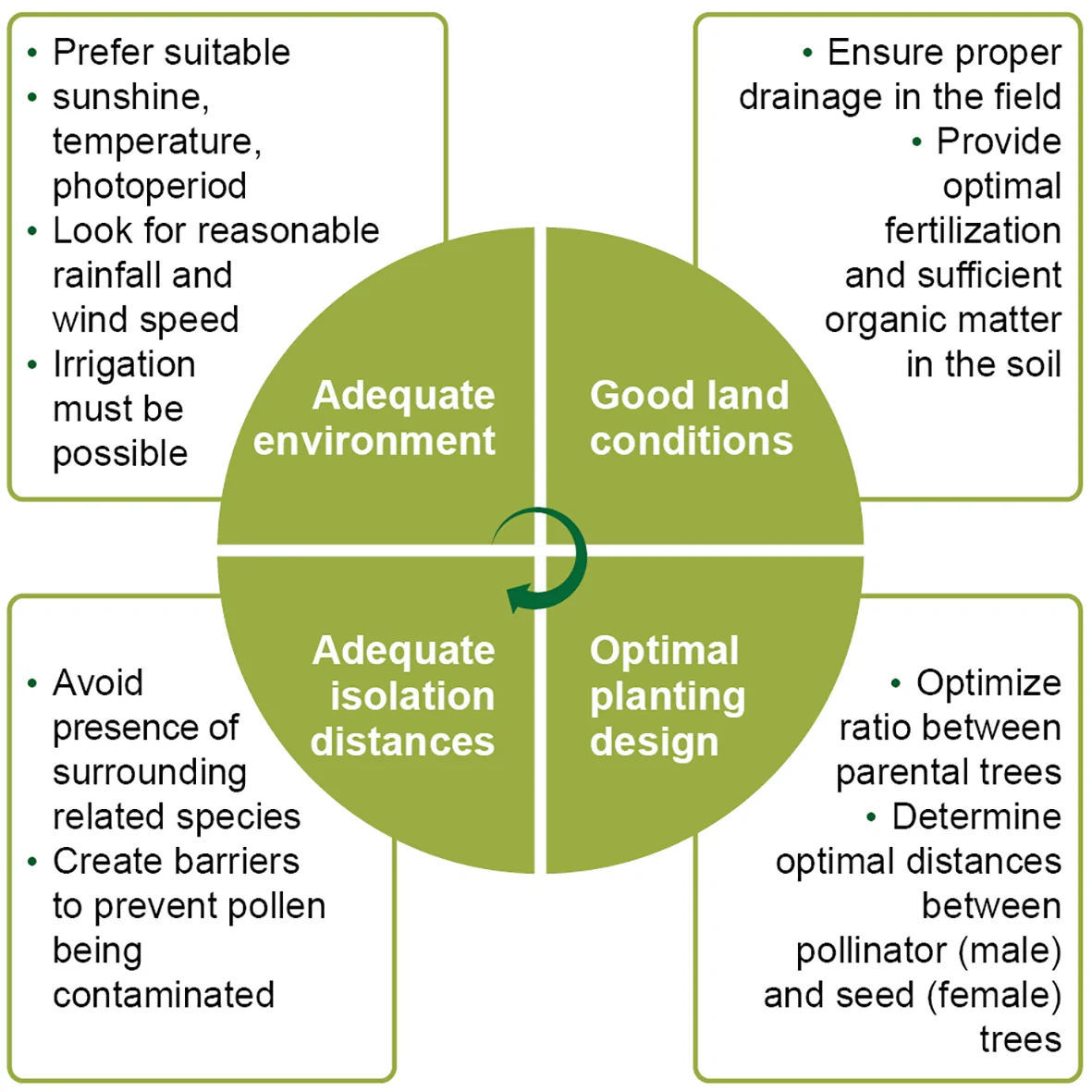
The agronomic conditions required to improve the efficiency of a seed production system for hybrid cultivars.

Among the factors that determine the final efficiency of a hybrid seed garden, some are linked to the agronomic conditions that determine the best establishment of the parent plants, as well as the optimal conditions required to maximize the efficiency of the pollination process (Perez-Prat and van Lookeren Campagne, 2002; Colombo and Galmarini, 2017). **Figure 4** shows the importance of external factors: an adequate environment and good land conditions, optimal planting material and adequate isolation distances, but also some field plot specifications like avoiding closeness to other related varieties through adequate isolation distances and respecting optimal inter-plant distances by using appropriate planting material, determine the overall efficiency of the seed garden.

As pointed out by Tiwari et al. (2024), to minimize the uncertainties in a seed garden system, breeders should conduct studies to identify the appropriate practices and conditions. Based on information collected in different research plots, seed production protocols could then be recommended to the seed production team. Concerning research, the proper window for sowing, staggered intervals, specific cultural practices such as nicking, sex modification techniques, and pollen preservation methods, play determining roles. Additional studies should investigate the specificities of the parental genotypes such as synchronized flowering (nicking time), and the seed yield potential of the female parent, among others. Furthermore, knowing the flowering behavior in terms of its initiation, peak flowering, termination and hence the duration of flowering, would allow an appropriate seeding/sowing plan of parental lines as well as possible adjustment of irrigation for each. Finally, another key factor is the optimal ratio of male-sterile (MS) female plants to fertile male plants, as an appropriate planting ratio ensures adequate pollen availability while maximizing the area devoted to seed production, thereby increasing F1 hybrid seed yield (Doddagoudar et al., 2006; Chakrabarty et al., 2023).

#### 4.1.3 Quality aspects in seed production

**Figure 5:**
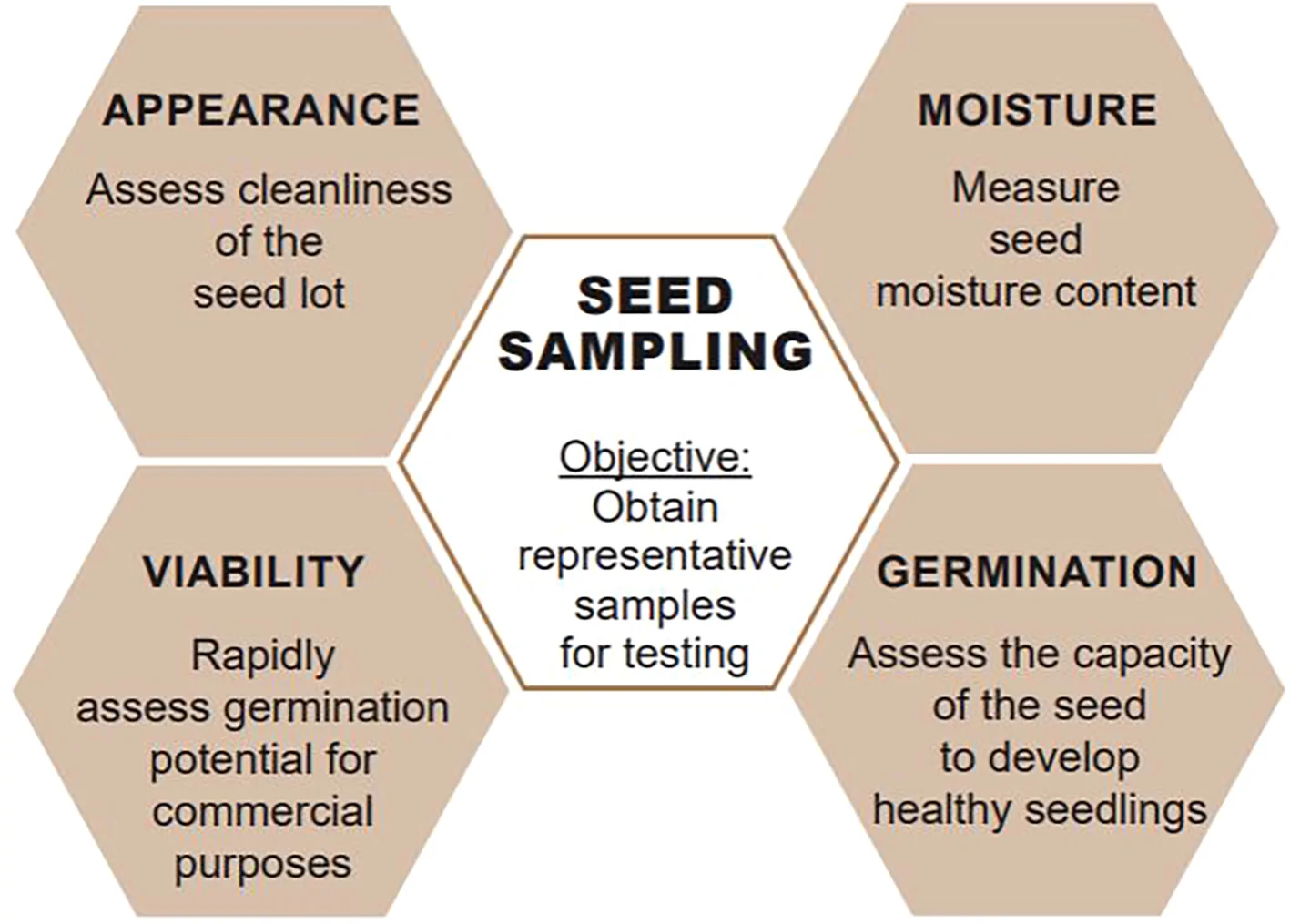
Main parameters that guarantee high quality standards for commercial seed production.

The value of a seed is intrinsically linked to its quality, making the evaluation of seed quality a critical component of any seed production system (Griffo et al., 2024). It is essential that seed quality is rigorously tested across all relevant parameters, adhering to established standards to be sure results are consistent and reproducible within acceptable tolerance limits. The main seed quality parameters are summarized in **Figure 5**. An adequate seed sampling procedure, i.e. using primary samples, is crucial to guarantee consistency and reproducibility through representative samples that are taken for laboratory analyses and tests. In commercial seed companies, the minimum number of primary samples depends on the size of the batch of seeds concerned, which ranges from 0.5 kg to 20 tons. Seed quality is determined by genetic purity, physical and physiological quality, which influences both germination potential and the suitability for medium-term storage, and the absence of contaminants. A guarantee that every seed is true to its type, implies the absence of contamination from other varieties or species, and is crucial for ensuring that crops are of the expected quality and agronomic performance. As recently reviewed by Hegde et al. (2025), image processing and machine learning techniques are increasingly employed for seed quality assessment to minimize human intervention while maximizing productivity in crop plant sector. Physiological quality influenced by a completed maturation process and optimum hydration results in good germination rates and seed viability while physical quality guarantees seeds are free from contaminants and exhibit minimal damage. The seed health status confirms the absence of pests and diseases. The quality of coffee F1 hybrid seeds should be tested based on all essential parameters applying standard procedures. For many important crops, the International Seed Testing Association (ISTA, https://www.seedtest.org/) plays a pivotal role in this process by developing and publishing standardized procedures for sampling and testing seeds, with the aim of achieving uniform seed testing worldwide (Milivojević et al., 2018). Physical purity assesses the cleanliness of batches of seeds, in terms of the chemical composition of the seed or external contaminants. Physical purity can be estimated by the frequency (%) of contaminants, i.e. inert particles, other seed species, tissues, as a function of the weight of the working sample.

Moisture content measurements provide insights into a seed’s current water conditions and seed viability that is directly influenced by the relative humidity of the storage room. The effect of water content on the longevity of *C. arabica* seeds is the subject of debate in the literature (Van der Vossen, 1977; Hong and Ellis, 1995). Indeed, it has been proposed that there are two optimum moisture contents for short-term storage of coffee seeds. A “low” optimum of moisture of 10% FW, and a “high” level around 40% FW (Dussert et al., 2012). According to the literature, the average L50 values observed for these two optimal water conditions are of the same order of magnitude, around 40 months. On the other hand, several observations made over many years in the field by CIRAD researchers showed that most coffee seeds should be stored between 20% to 30% FW and that maximum optimal storage is around 18 months in cold-room at 15-18°C, for maintaining seed germination capacity without significant deterioration (unpublished observations).

In addition, germination tests evaluate the coffee seeds’ capacity to produce healthy and vigorous seedlings. The germination capabilities of a seed lot involve different stages and physiological mechanisms that can differ from one species or variety to another (Castanheira Guimarães et al., 2013; Tristão Alixandre et al., 2021; Fantazzini et al., 2022). Germination tests should include moisture, aeration, temperature, and light conditions. Rapid methods for assessing coffee seed viability complement these evaluations (Hilst et al., 2016). Rapid methods based on staining solutions are available for the assessment of seed viability. Other parameters related to seed viability are seed health and seed vigor. Collectively, these quality control measures are crucial to increase commercial seed productivity and ensure high quality standards for present and future F1 Arabica seeds. High-quality seeds are integral to climate-smart agriculture, contributing to significant increases (up to 15-20%) in crop productivity and supporting sustainability goals in many crops (Tiwari and Park, 2024). Therefore, producing high quality hybrid seeds in well managed coffee seed gardens would drastically improve agricultural resilience in the face of climate change.

### 4.2 The main technical aspects for establishing seed gardens to produce Arabica F1 hybrid seeds

**Figure 6:**
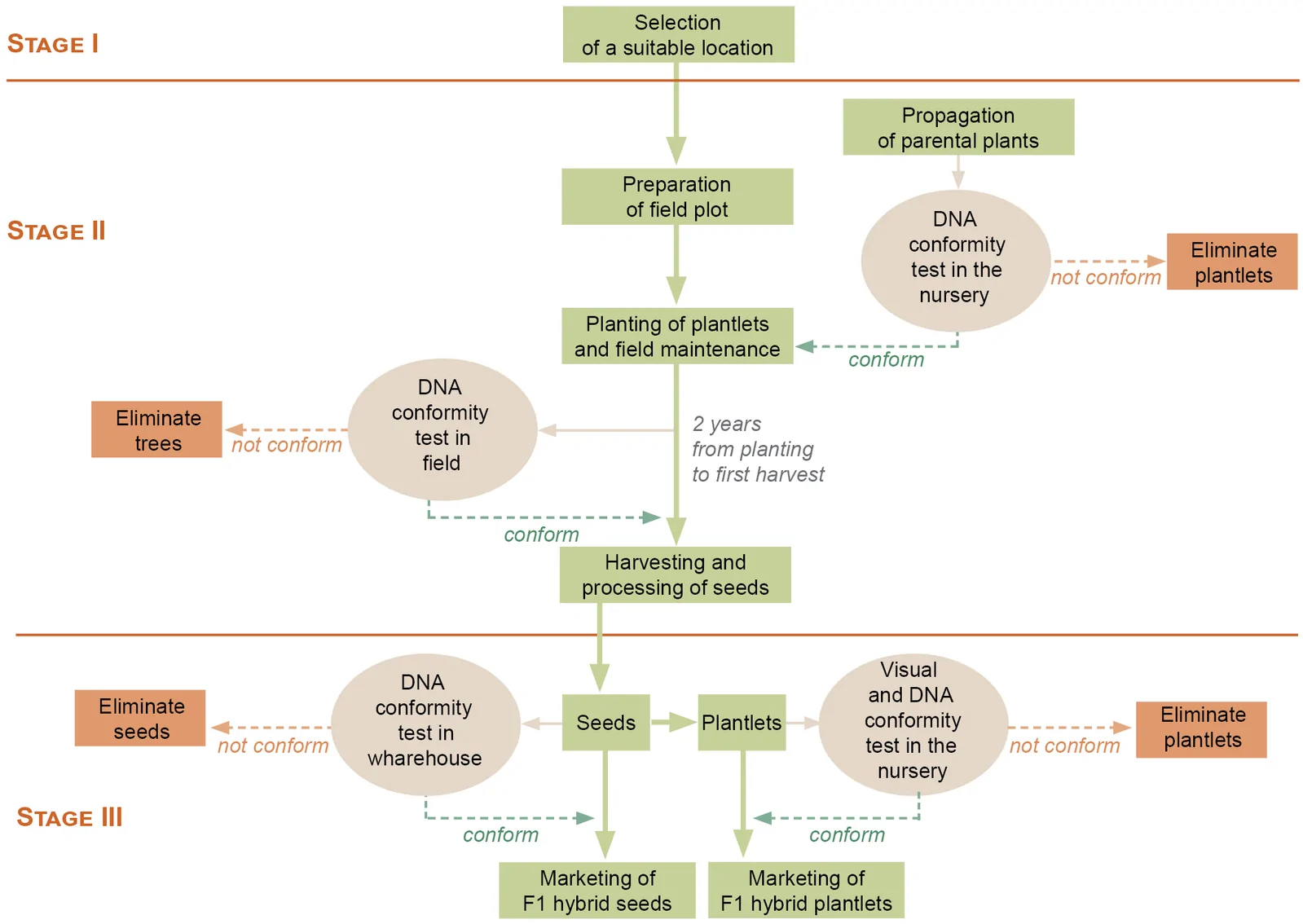
Stages required for the correct implementation of a seed garden for Arabica hybrids. Critical time points at which DNA conformity tests should be conducted are necessary to ensure the quality of the final products (seeds and/or plantlets).

Based on the considerable experience already gained on vegetable crop species but also gained by CIRAD-ECOM collaboration and by Nestlé company specifically on coffee F1 hybrid seed garden implementation (Georget et al., 2019), the following section reviews some of the main technical aspects that need to be taken into consideration when implementing large scale Arabica F1 hybrid seed production using male sterility. The main stages in the establishment of seed gardens in coffee are summarized in **Figure 6**.

#### 4.2.1 Site selection criteria to respect when establishing coffee seed gardens

Ensuring that the field growing conditions of the two parental lines are adequate in terms of latitude, altitude, and temperature is the first requirement to fulfil for the rapid establishment of a seed garden. Medium altitude areas (700-1,000 m) are preferred to high (>1,200 m) or low areas (<500 m) (with high temperatures) where weather conditions would be extreme and hence limit optimal flowering and fruit production. Furthermore, it is strongly recommended to choose a location with a clearly marked dry season (3–5 months per year), so that the main flowering events only take place once or at the most, twice a year. These conditions facilitate massive and synchronous flowering events while still offering the possibility to use controlled irrigation to induce additional flowering events to avoid foreign pollen contamination. Regulated shading is also a critical factor for satisfactory plant establishment during the first two years following planting, but also during the reproductive season especially in areas where the intensity of the sun could affect optimal maturation of the cherries. In addition, choosing moderately flat locations will avoid excess drainage while simultaneously facilitating maintenance and harvesting operations.

#### 4.2.2 Aspects related to the multiplication of the parental lines

**Figure 7:**
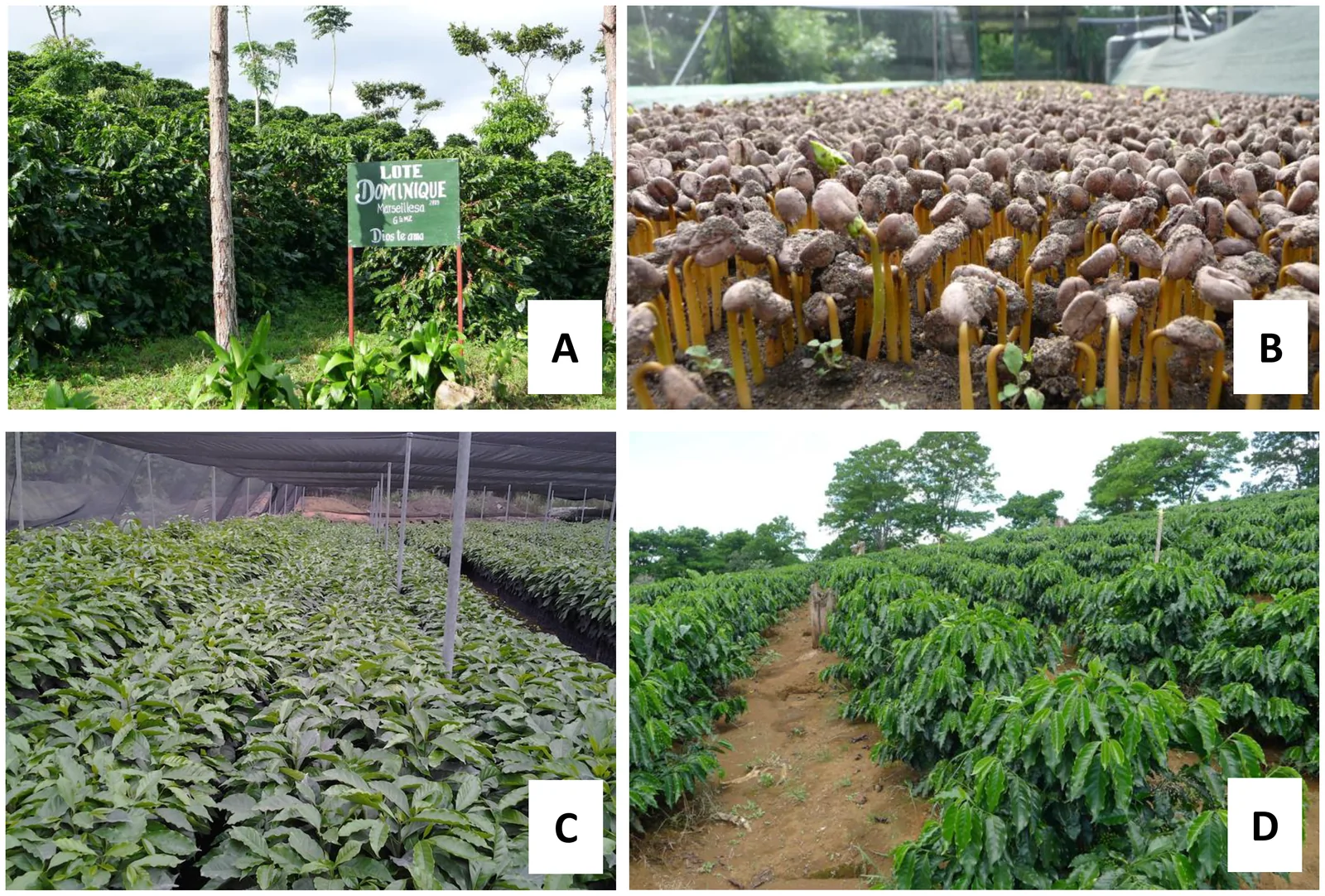
Origin and deployment of pollen donors in a seed garden. **(A)** Plot of a pure line variety of pollinator **(B)** Germination of certified seeds of a variety of pollinator **(C)** Screening of the off-type plants at the nursery stage **(D)** Seed garden planted with a genetically verified pure line variety to be used as donor pollen to produce F1 hybrid seeds.

Some specific requirements concern the multiplication and the compliance of the parental lines as illustrated in **Figure 7**. Indeed, in coffee the pollinator parent is usually propagated locally using seeds in the country where a certified seed garden has been established under the auspices of the National Coffee Research Centre in order to ensure the genetic conformity of the pollinator, the pollinator being produced by controlled self-pollination, i.e. by protecting flower buds from other contaminants. This ensures the homogeneity of the pollinator seedlings. To avoid any cross pollination, the pollinator can also be produced by asexual way thought somatic embryogenesis or by horticultural way (Georget et al., 2017; Etienne et al., 2018).

**Figure 8:**
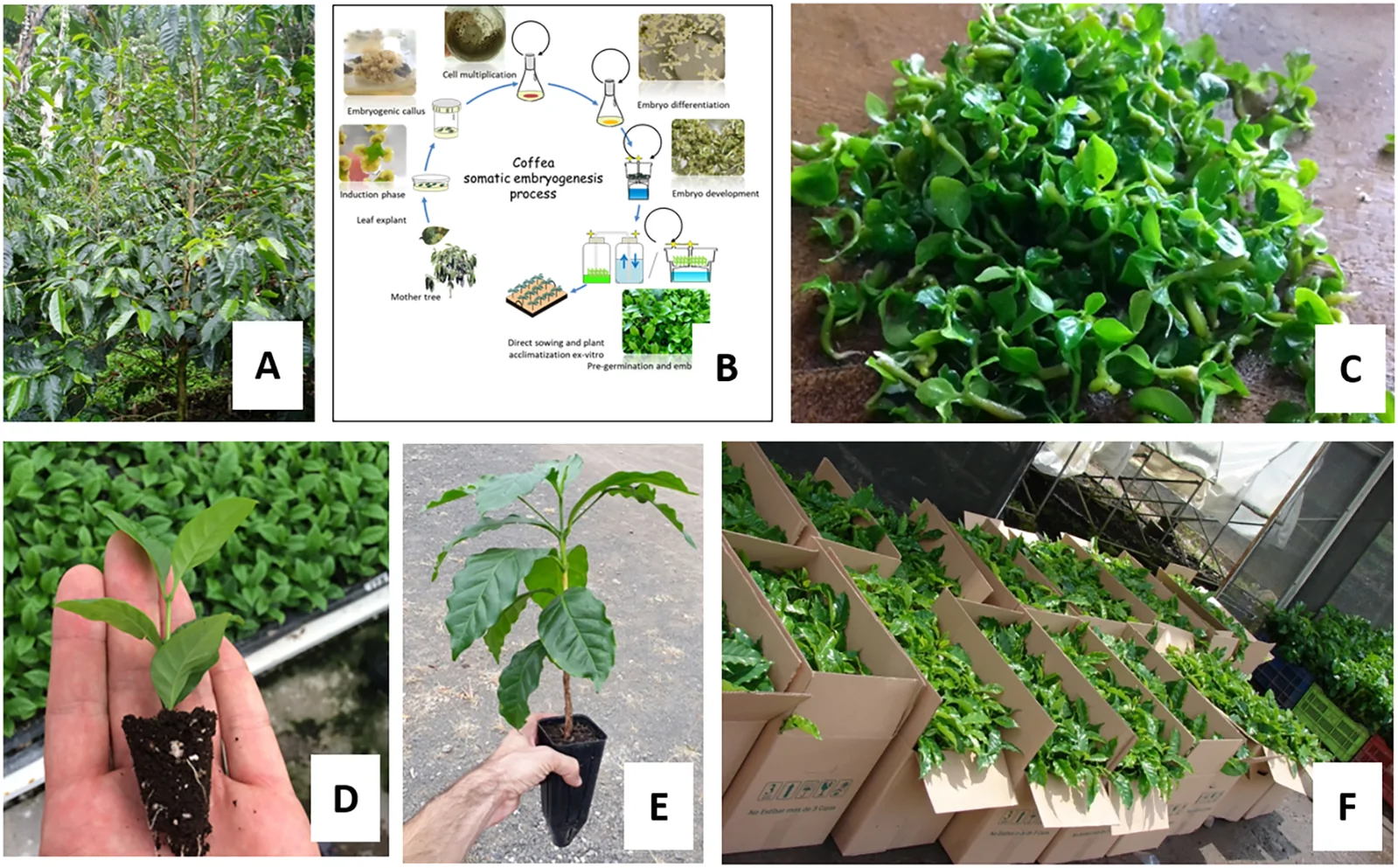
Production of MS plants to establish seed gardens. **(A)** MS plant accession from the Ethiopian collection **(B)** MS propagated using the somatic embryogenesis (SE) technique **(C)** Somatic embryos of the MS variety **(D)** 8-weeks-old acclimatized MS plantlets obtained after embryo acclimation in the greenhouse **(E, F)** 6-months-old MS plant obtained in the nursery ready to be transported and planted in the field.

Currently, the male sterile parent is propagated by somatic embryogenesis before being shipped to the target country for acclimatization and hardening process in nursery (**Figure 8**). Importing these plantlets requires an import permit which should be applied for in advance. To this end, an official request from the seed grower should be sent to the company that owns the plant material. The seed grower must make sure that both parental plants are at a similar stage of development before planting in order to ensure synchronized development of the F1 seed garden. If this is not the case, a cycle of rooted mini cuttings (Georget et al., 2017) will be needed to homogenize the size of the plant material before planting the seed garden.

#### 4.2.3 Physical isolation requirements

**Figure 9:**
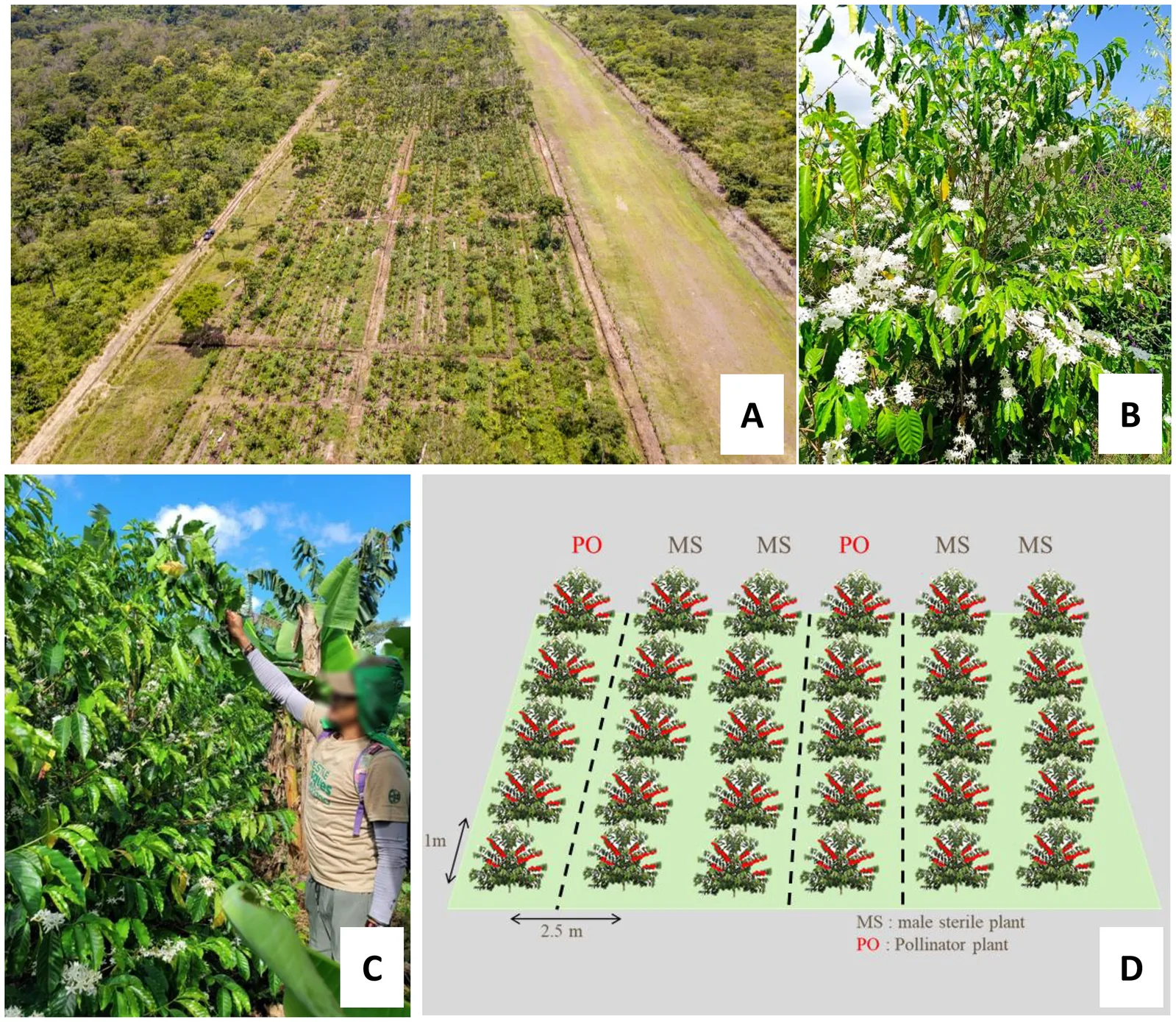
Set up of a 6-ha F1 seed garden in Nicaragua on ‘La Cumplida’ farm isolation requirements and flowering/pollination management. **(A)** Aerial view of the seed garden **(B)** Flowering synchronization of MS plants, **(C)** Manual pollination of MS plants using detached pollinator branches **(D)** Schematic distribution of parental genotypes in a hybrid seed garden. Male sterile plants are planted in two rows followed by one row of pollinators (pollen donor parents). Suggested distances between plants and between rows are shown.

When a seed garden is established in a coffee growing area, a minimum distance around the seed garden should be defined within which coffee should not be cultivated (**Figure 9A**). Arabica pollen is quite heavy (Chong et al., 2014) and has a flying range of around 100 m, hence, the minimum distance required for good isolation is estimated to be 200 m in all directions, even if in shaded coffee landscapes it has been reported that some native bees can pollinate up to a distance of 1,800 m (Jha and Dick, 2010). Natural barriers planted around the parcel are a complementary strategy to reduce pollen contamination. These natural barriers can be bushes or trees that surround the seed garden. The best option is to plant a double line of pollinators as a barrier round the entire seed plot as it ensures isolation and provides additional pollen to the MS plants.

#### 4.2.4 Flowering and pollination management

Coffee flowering can be concentrated by irrigating after a relatively long dry period (Rochi et al., 2015; Rendón-Sáenz et al, 2025). Indeed, floral synchrony in coffee occurs because dormant buds do not advance to the next stage during dry periods, allowing buds at an earlier developmental stage to also reach dormancy and achieve more uniform flowering once the rehydrating effect of the first rains or irrigation occurs. Is this water stress that promotes greater synchrony of floral opening of the buds in a dormant state. From a practical point of view, irrigating when more than 60% of flowers reach the ready-to-open stage, makes it possible to synchronize the intensity of flowering in seed gardens where seed production is the priority (**Figure 9B**). Therefore, in the dry season, it is recommended to induce uniform flowering by supplying enough water to the coffee plants, i.e. about 100 L/adult plant. By artificially advancing flowering, the exchange of pollen between trees belonging to neighboring commercial plots is prevented. The precondition for this arrangement is that the plot is equipped in advance with an efficient irrigation system and that sufficient water resources are available near the plot for the purpose of irrigation.

The conditions that allow the dissemination of pollen in the seed garden to enhance cross pollination between the two parental lines should be promoted and implemented if required. In that sense, using ventilators or leaf-blowers will encourage cross-pollination, especially if the wind flow on the plot is reduced at the time of flowering. Bringing beehives to the plot during flowering can also facilitate cross-pollination (Hipólito et al., 2020). However, the beehive option should only be implemented if the seed garden is well isolated from other coffee plots or if flowering was induced by irrigation during the dry season when the other plots were not yet in flower. As reported for other crops such as rice (Chakrabarty et al., 2023), an alternative method is supplementary pollination performed by manually shaking pollen donor branches over the male-sterile plant once flowering occurs to facilitate cross-pollination (**Figure 9C**). Because freshly opened flowers only remain receptive for a very short period (a few hours up to maximum two days) before degenerating, shaking campaigns need to be well synchronized. Field observations showed that this simple technique increased the efficiency of coffee plant pollination by a factor of 15% to 20%, especially in the absence of wind (unpublished observations).

In addition to biotic and abiotic factors already mentioned, planting system (i.e. spatial arrangement of the parental plants) in the seed garden is an important factor affecting the efficiency of cross-pollination. Therefore, the two parental lines should be planted following a specific design which will need to be established for each species using an experimental trial-and-error approach. For example, to produce the nematode-resistant *C. canephora* rootstock hybrid variety known as Nemaya, the seed gardens established throughout Central America alternated one row of each parent plant (Bertrand et al., 2002). Different planting patterns are possible, but the final volumes of seeds will mainly depend on the number of male sterile plants in the plot. To increase the productivity of Arabica F1 hybrid seeds, the 2:1 pattern is recommended, although other planting designs are possible (**Figure 9D**). In the 2:1 design, each row of pollen donor is followed by two rows of male sterile plants. To ensure the availability of pollen during flowering, the plot should be bordered by pollinator trees in a single or double row. In all cases, both the rows of male sterile plants and the rows of pollen donors should be identified with labels or using a colour system to avoid any confusion during harvest. With a planting density of 4,000 plants/ha, one hectare of seed garden will require 2,667 male sterile and 1,333 pollinator plants. The distance between the plants in a row should be 1 m and the distance between the rows should be 2.5 m. However, this distance will differ from one location to another depending on the temperature and the altitude of the parcel. In medium-low altitudes, plants will grow faster, so the distances between plants should be greater (1.5 – 1.8 m between plants in a row). If necessary, planting distances should also be adapted to mechanization, which enables a significant reduction in production costs. Contrarywise, low densities of parent plants could reduce final volumes of seeds due to inadequate pollination conditions.

### 4.3 Coffee F1 hybrid seed harvest, processing and quality control

#### 4.3.1 Improving the seed harvest

**Figure 10:**
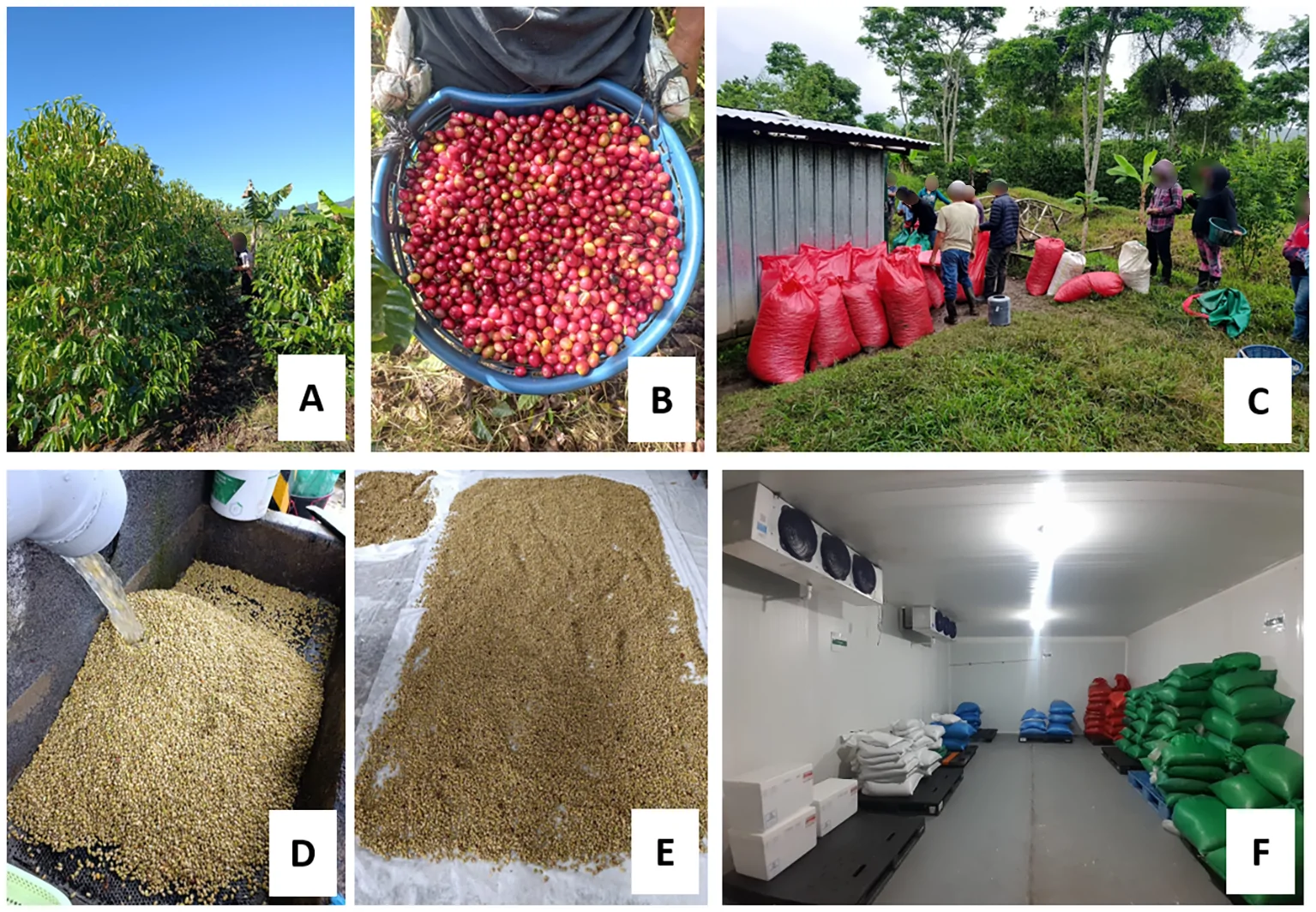
Harvesting, processing and storage of F1 hybrid seeds. **(A)** Harvesting of the seed garden **(B, C)** Fully ripe cherries are harvested and placed in different colored bags that are easily distinguishable from the other bags used on the farm. **(D)** Washing of the cherries. **(E)** Drying seeds in a shed. **(F)** F1 seeds stored in bags in a temperature-controlled room (15°C–18°C with 50% RH).

Cherries from the female (MS) and male parents (P) have to be harvested separately. The cherries harvested from the female parent contain the hybrid seeds and are the result of the MS x P cross. The cherries harvested from the male parent can be used either to produce green coffee or to produce certified seeds of the pollen donor variety. These seeds are indeed the result of a P x P cross and are therefore genetically pure and homozygous. Particular care should be taken to ensure that the MS cherries are harvested when fully ripe (red-violet in colour) and placed in bags of a colour that is easily distinguishable from the other bags used on the farm, to avoid the possibility of the fruit getting mixed at the processing unit (**Figures 10A–C**). Systematic labeling of the bags is also strongly recommended to guarantee traceability. Labeling should contain key information such as the plot code, MS tree genotype, and the harvest date.

#### 4.3.2 Post-harvest and storage adaptations

Before pulping hybrid cherries, the pulping equipment must be carefully calibrated to avoid mechanical damage to the embryo inside the seed. Even if it has been suggested that motorized pulper can be used to process coffee seeds in large quantities with less time, labor inputs and good results (Adeleke et al, 2023), manual pulping remains the preferred method for seed production. As the coffee embryo is located in the superficial zone of each cotyledon (Etienne et al., 2013), an injury caused by the pulping can cause malformation of the root or even the death of the embryo. After pulping, the coffee is at the parchment stage, but is still covered with the remains of the pulp and must therefore be washed before fermentation to remove all traces of mucilage (**Figure 10D**).

The duration of the fermentation process will vary with the climatic conditions of the farm, nevertheless it is recommended that fermentation not exceed 16 hours (Brando and Brando, 2014). When possible, it is recommended to wash the pulped coffee in washing channels, which facilitates not only the removal of the mucilage but also enables complete separation from the empty beans and the impurities.

Drying the seeds is one of the most critical stages of the process, since it reduces the amount of water in the cotyledons to the minimum required to guarantee the viability of the embryo and its storage in the best conditions (Dussert et al., 2012). In order to ensure slow and homogeneous drying, it is highly recommended to dry the seed under canopies protected from direct sunlight using an 80% shade net or in a shed (**Figure 10E**). The final packaging must guarantee the seed is protected from both light and moisture. Therefore, it is recommended to pack each batch of seeds in high-strength polyethylene bags. Each bag should be labeled with all the data needed to check its identity, thereby guaranteeing traceability. A warehouse located close to the seed garden is needed to store the seeds after processing (**Figure 10F**). The cold room should be maintained at 15 -18°C with 45-50% of ambient relative humidity.

#### 4.3.3 Quality control

Before establishing a new seed garden, it is the duty of the company that owns the parental plant material to conduct conformity tests on the parental plantlets before delivering them to the seed grower to ensure that the progeny (seeds or plantlets) will have the expected genotype (Anteneh et al., 2014). In the case of coffee F1 hybrids, each batch of fresh seeds should undergo a series of quality evaluations to check key parameters, as already mentioned, i.e. clean appearance, good viability, high germination rate and proven genetic identity.

The appearance of the seed is focused on parchment samples that should be even amber yellow in colour. There should be no visible signs of insect or mechanical damage. The seeds should also have no trace of fungal infestation. The frequency of signs of poor seed quality - in appearance or in health - should in no case exceed 1% of the batch.

Information concerning the identity of each seed batch should use standard traceability methods as well as precise tests to guarantee the right genetic origin. Conformity tests based on molecular markers, mostly single sequence repeats (SSRs) have been used in Arabica coffee to precisely trace genetic origin, excluding any off-type seeds that are the result of external pollen contamination (Georget et al., 2019; Pruvot-Woehl et al., 2020). According to UPOV, the thresholds for permitted off-types vary between 0.5% and 2%, they may be stricter (like for autogamous cereals), or on the contrary, more flexible for complex genetic hybrids as is the case for the 3-way coffee hybrid Ruiru 11 produced in Kenya (Opilé and Agwanda, 1993) or even hybrids using male sterility systems that require the mixture of the female parent and the pollinator to be marketed as a variety. The viability of the seed is a direct indicator of the final quality of the seed produced. The good viability of the seed guarantees adequate germination potential, and consequently more healthy and vigorous seedlings for the farmers (Rosa et al., 2011). In the end, the percentage of fresh seeds that germinate depends on using good practices throughout the entire process from harvesting to drying and storage. In addition, there is evidence showing that varietal differences in coffee significantly influence seed germination, vigor, uniformity and growth performance (Rago and Tamasgen, 2025). Under good drying and storage conditions, *C. arabica* seeds remain viable for at least one year and *C. canephora* seeds for six to eight months (Couturon, 1980; Hong and Ellis, 1995; Dussert et al., 2012). Seeds destined for commercial coffee propagation should have a minimum of 85% of germination and between 20-25% of humidity. This percentage is determined by a germination test, which must be carried out on each seed batch 30 days after germination. After this period, the seed begins to lose viability, regardless of the storage conditions (Rosa et al., 2011).

## 5 Challenges and opportunities for large-scale dissemination and adoption of coffee F1 hybrids

Many case studies of different crops demonstrate the success of hybrids, nevertheless, different technical, environmental, and socio-economic constraints in scalability and accessibility persist (Thakur et al, 2026). While the objectives of coffee breeding have changed over the last two decades, 25 years of experience in the dissemination of modern coffee F1 hybrid varieties in Central America has shown that the adoption of F1 hybrids is strongly influenced by a number of challenges related to the higher cost of improved material, access to planting material and uncertainty due to the lack of information concerning the performance of the variety among farmers as well as among coffee buyers (Turreira-Garcia, 2022; Kahsay et al., 2023). Since smallholders represent by far the largest number of coffee farmers and a substantial percentage of coffee production, it is vital to facilitate their access to F1 hybrids. A variety of strategies have been in recent decades to address these constraints. The following paragraphs provide an update on the situation in 2026.

### 5.1 It is now possible to remove economic constraints

Several implementation strategies could be proposed to reduce the cost of Arabica F1 hybrid produced by clonal propagation and facilitate their wider dissemination. These following approaches were supported by experiences in specific contexts but have not yet been systematically validated across a wide range of production systems or countries. One of them would be to entrust the production of F1 Arabica hybrids to national coffee institutes that can receive specific funds from governments to promote the renewal of coffee plantations and would hence be able to sell the plants to the farmers at cost price. In this case, political choices must be made at the national level to facilitate the purchase of plants by farmers either by distributing the plants free of charge, or allowing the farmer access to the plant material through loans at preferential rates. The lack of access to credit has been reported to be the main barrier to the dissemination of modern improved coffee material for a number of years, especially among smallholder farmers (Van Der Vossen et al., 2015; Ward et al., 2017). Another alternative would be to entrust large scale plant production to private companies in the coffee sector, i.e. traders or roasters. This was the case for example in Nicaragua where since 2004, the ECOM group invested in collaboration with CIRAD, in a tissue culture lab and sophisticated nurseries to produce Arabica F1 hybrid plants by clonal propagation. To date, the ECOM/CIRAD alliance has produced around 30 million F1 Arabica plants by clonal propagation in Nicaragua, representing around 10,000 hectares of coffee plantations or 6% of Nicaragua’s coffee-growing area (Etienne et al., 2025).

Another proposed implementation strategy would be to entrust large-scale plant production to professional horticulturists. In that case, the cost could be reduced by industrializing the SE process or other vegetative propagation processes for example, *in vitro* micro-cuttings or horticultural rooted mini-cuttings. However, the question of financial profitability remains given the currently low prices of coffee plants that should not exceed 0.5 US$ for a 6-month-old plant in nursery bag ready to be planted in the field, which represents a huge challenge.

In remote places where access is limited, the production of F1 Arabica plants can be entrusted to cooperatives or to farmers themselves. Inspired by farmers’ seed systems in Africa (Magesa et al., 2013) that are mainly suitable for reproduction by seed, a cooperative seed network was set up in 2017 in Nicaragua in the framework of the EU BREEDCAFS project (https://cordis.europa.eu/project/id/727934). The horticultural rooted mini-cuttings technique was made available to women’s cooperatives in order to reduce the higher cost of F1 Arabica plant compared to that of vitro-plants directly produced in the laboratory by somatic embryogenesis and also to reduce gender inequalities (Etienne et al., 2018). This approach has been implemented in three contrasting production contexts (Vietnam, Cameroon and Nicaragua), providing encouraging initial results, starting with a small quantity of initial somatic seedlings supplied by a certified seed producer and renewed every two years. Today all the nurseries involved are still propagating Arabica F1 hybrids by rooted mini-cuttings. Batches of *in vitro*-plantlets are dispatched by air to the destination country and further acclimatized and multiplied by a factor 20 to 40 for year via mini-rooted cuttings. The women’s cooperatives reproduce and market the plants derived from rooted mini-cuttings that are distributed to local growers. The cooperatives also agree to pay an annual royalty that recognizes the breeder’s rights. This mechanism is guaranteed by the coffee buyer at the same time as an Agroforestry business driven cluster with Arabica F1 hybrids is set up. This alternative has the advantage of providing a certain degree of autonomy in the production of hybrids at the regional level, but the cost price remains still higher than that of seedlings of inbred varieties.

Finally, seed production based on male sterility represents a promising pathway for large-scale dissemination of F1 hybrids at reduced cost. This approach has the potential to improve access to Arabica F1 hybrids for smallholder coffee farmers who cannot afford to invest in Arabica F1 hybrid plants produced by clonal propagation, although its large-scale commercial validation across diverse production systems is still ongoing. However, the initial investment in research and development required to implement this kind of production system is high and should not be underestimated.

### 5.2 Increasing smallholders’ access to F1 hybrids

Several solutions have been put forward to increase access to F1 hybrid production, for example, decentralizing production by establishing networks of nurseries near coffee-growing areas or by integrating hybrid multiplication based on rooted mini-cuttings into existing local nursery infrastructure (Georget et al., 2017). New advances in seed-based hybrid propagation have made it possible to remove most of the obstacles to their adoption including larger scale dissemination at low cost and easy access to coffee F1 hybrids for the farmers.

Another major barrier to the dissemination of elite coffee material is limited access to credit, particularly for smallholder farmers seeking to renovate their plantations with improved varieties (Van Der Vossen et al., 2015; Ward et al., 2017). To date, Costa Rica has been the most successful in adopting F1 hybrids, partly due to relatively high farm-gate prices and easier access to credit compared to many other producing countries. This highlights the critical role of international coffee prices in the diffusion of new varieties. Additionally, Costa Rica benefits from a strong reputation and recognized coffee quality, meaning it can demand high premium prices.

Even when farmers are able to buy coffee plants near their farm and have the financial capacity to invest in F1 hybrid plants, lack of knowledge may persist regarding the agronomic performance of the new varieties under local agroecological conditions and access to premium markets. Such uncertainty strongly influences farmers’ choice of varieties, as they are often risk averse given the importance of stable coffee income for their livelihoods (Jezeer et al., 2019). In addition, uncertainty about the cup quality of new varieties may discourage some coffee buyers and roasters from purchasing these coffees (Van der Vossen, 2009). For farmers, traders, and roasters alike, decisions related to the adoption of new cultivars must therefore rely on robust scientific knowledge of the plant material, particularly genetic, agronomic, cup quality and environmental interactions that are key factors for varietal improvement (Montagnon et al., 2000, 2008; Ahmadi et al., 2013). Although scientific studies are often available, farmers are rarely the primary target audience. When results are not widely shared with the broader coffee community but are limited to specific actors, lack of trust can become a barrier to the adoption of new plant varieties, even when their performance is well documented (Reinker and Gralla, 2018). Finally, farmers’ conservatism may represent a significant barrier to the adoption of new coffee varieties. Many growers prefer to continue cultivating the varieties they have relied on their farm for decades and are often reluctant to embrace changes. This behavioural factor should not be underestimated, as it can substantially slow the diffusion of improved coffee varieties, even when these offer clear agronomic or economic advantages (Turreira-Garcia, 2022).

## 6 Future trends and research priorities

### 6.1 Improving the efficiency of hybrid F1 seed gardens

Optimizing pollination efficiency and developing specific agronomic protocols adapted to each environment will increase seed yield and reduce costs. For example, in Nicaragua, the yield of seed gardens was significantly increased - from 0.5 to 1 ton of hybrid seeds per hectare - improving flowering synchronization by equipping each parental line with its own irrigation network, combining natural cross pollination with semi-manual pollination using some branches taken from the male parent line and shacked above the sterile male plant during the flowering period, and optimizing management and nutrition of the genitors though fertilization and adequate tree pruning (preliminary field observations). Advances in genomics and quantitative genetics will also support more precise selection of parental lines, improved male sterility systems, and enhanced synchronization of flowering.

### 6.2 Integrating clonal and seed-based methods in F1 hybrid dissemination strategies

Clonal propagation using a combination of somatic embryogenesis process/rooted-mini-cuttings and seed-based propagation is complementary. Clonal propagation ensures its genetic structure is identical to that of the selected individual F1 plant and is ideal for high-value or specific markets while seed propagation, although it produces offspring from the same two parents with a certain degree of genetic variability, nevertheless increases accessibility and facilitates large-scale dissemination. Strategies combining the two methods will optimize deployment to meet regional needs and the target market. The choice of one type of material over another will depend on different criteria: (i) demand for the varieties by coffee actors, be they farmers, traders, roasters or consumers; (ii) their agronomic performance in the target country; (iii) the farmers’ experience of the technology and the profitability of the variety; (iv) the coffee cultivation system targeted, e.g. low or high use of fertilizer, agroforestry vs full sun; (v) farmers’ access to plant material; (vi) the target market, i.e. mainstream or specialty coffee; (vi) and sales price of improved material.

Finally, the country’s economic and social condition also play an important role in the farmer’s decision and acceptance of a given variety. Interestingly, in Ethiopia, risk vulnerable farmers preferred seeds adapted to their local conditions and coffee varieties that produced a stable yield, whereas farmers in more accessible areas and/or those who are less concerned in securing subsistence income levels opt for attributes that maximize income, namely, yield and marketability (Wale et al., 2005). It is consequently important to incorporate several types of new seeds and clonal material into the dissemination strategy as well as to propose a panel of several coffee varieties with contrasted genetic and agronomic characteristics to allow further selection of the most suitable varieties in accordance with farmer’s preferences and the local context.

### 6.3 New strategies to encourage farmers to adopt Arabica F1 hybrids

**Figure 11:**
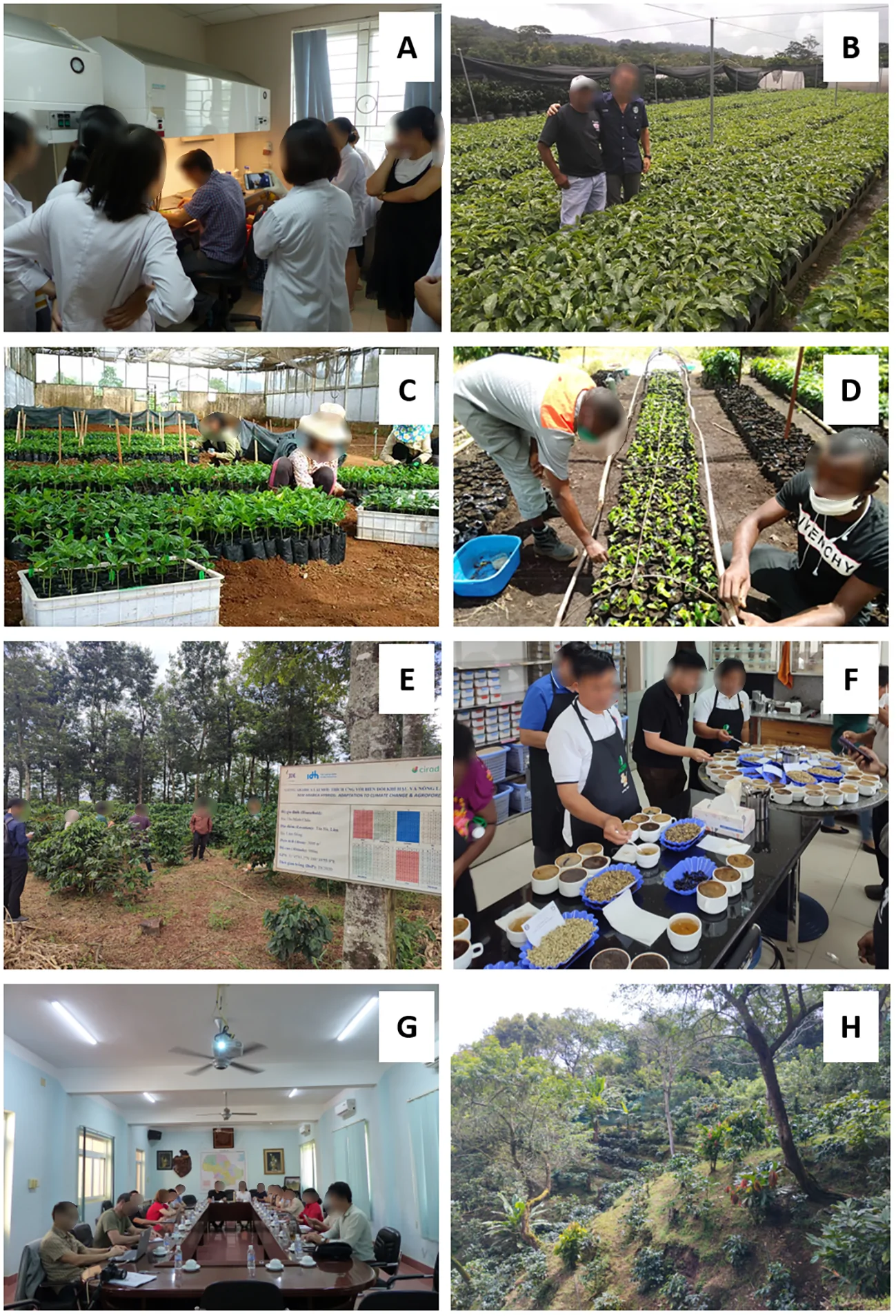
New strategies to increase dissemination and farmers’ adoption of new coffee hybrids (European BREEDCAFS projects 2019-2021). **(A)** Transfer of SE technology to AGI (Agricultural Genetics Institute) in Vietnam. **(B–D)** Transfer of rooted mini-cuttings technique in Nicaragua, Vietnam and Cameroun. **(E)** Implementation of on-farm demonstration plots in Vietnam. **(F)** Assessment of cup quality of coffee grown in on-farm demonstration plots. **(G)** Implementation of dialog platform in Vietnam. **(H)** Set up of small-scale business-driven agroforestry clusters with F1 hybrid varieties in Nicaragua.

In the framework of the European project named BREEDCAFS (2019-2021) a new dissemination strategy was applied in Nicaragua, Cameroon and Vietnam to accelerate the adoption of Arabica F1 hybrids by farmers (Etienne et al., 2018, 2025). The overall approach is based on multidisciplinary and public-private research collaboration, and has seven pillars: (i) technology transfer of the clonal propagation methods such as for example somatic embryogenesis (**Figure 11A**) and horticultural propagation techniques to national research institutions and women’s cooperatives to create autonomy to enable the countries to propagate the new varieties themselves (**Figures 11B–D**); (ii) establish on-farm demonstration plots among small scale farmers to enable them to assess the agronomic performance of F1 hybrids, cup quality, i.e. chemical and sensory data, and collect farm records for productivity and profitability analyses (**Figures 11E, F**); (iii) conduct surveys among farmers concerning their own perceptions of the new hybrids compared with local varieties and the main challenges related to the assessment and adoption of varieties. Implementing participatory methods of breeding selection has been shown to improve the adoption of coffee F1 hybrids by coffee farmers (Turreira-Garcia, 2022; Kahsay et al., 2023); (iv) set up and operate national dialogue platforms with all stakeholders to enable sharing of results and the exchange of knowledge concerning F1 hybrids and their production in agroforestry systems (**Figure 11G**); (v) establish initial, small-scale business-driven agroforestry clusters with F1 hybrid varieties including farmers and potential buyers (**Figure 11H**), and (vi) register the best candidate varieties in selected countries before beginning large-scale production.

The future of large-scale production and dissemination of F1 hybrids also depends on creating strong local production hubs, reducing reliance on international transport of plant material, and fostering public–private partnerships. Training local nurserymen in coffee propagation methods and establishing regional seed production centers will play a key role in their sustainable adoption. As pointed out by Lambot and Herrera (2018), any new propagation strategy in coffee should be economically viable for the agro-seed industry, as this is a precondition to maintain the competitiveness of the coffee crop compared with other industrial crops including rubber, and oil palm, for which genetic innovations are increasing faster than in orphan crops like coffee.

## 7 Conclusion

Coffee F1 hybrid varieties are a major innovation to rapidly establish climate-resilient and sustainable Arabica production systems. These varieties, which are more vigorous and resilient than conventional varieties, can be selected rapidly, i.e. in 10 versus 30 years and in greater numbers, making it possible to offer a wider range of varieties. Advances in clonal and seed-based propagation techniques now enable their large-scale dissemination. While significant technical, economic, and organizational challenges remain, hybrid production using seeds already has the potential to transform global coffee cultivation by accelerating genetic progress, improving farmers’ access to the best varieties, and fostering a climate-smart transition across coffee producing regions. In this paper, we have proposed some solutions to enable the large-scale dissemination and adoption of these hybrids worldwide. While these solutions can still be optimized, they already represent significant progress, especially given that no seed industry is currently fully invested in developing coffee varieties, multi-location validation, seed production and marketing.

A critical issue is how to finance breeding. Indeed, given the accelerating pace of climate change, there will be a continuous need to develop new F1 hybrid coffee varieties adapted to increasingly diverse but also changing climatic conditions. However, the seed industry is likely to invest in breeding only if seed production becomes economically viable, which is not necessarily the case at present considering the low sales prices of coffee plants in nurseries worldwide, approximately US$ 0.5–0.8 for a six-month-old plant in a planting bag.

This situation suggests that agricultural policy decisions will likely be required at the national level to facilitate farmers’ access to improved planting material. Such measures could include public subsidies or dedicated credit schemes for renovating coffee plantations at preferential rates of interest to encourage the adoption of improved varieties. Improved product valorization, through the development of market-oriented concepts such as the “AFS cluster business-driven” approach described above, would also enable farmers to achieve higher farm profitability and, consequently, invest more easily in the renovation of their coffee orchards.

Another challenge is to make more sterile male plants available in order to increase the number of possible hybrid combinations. The search for natural mutants in collections or plantations has been the preferred approach to date, but remains laborious and uncertain. Compared to the effort the different institutes involved in coffee breeding invest, the number of sterile male mutants discovered that are suitable parents for the creation of F1 hybrids remains extremely low. The recent development of genome editing techniques in coffee plants (Breitler et al., 2018, Casarin et al., 2022, Etienne et al., 2025) using the CRISPR technique appears to be a promising way to induce male sterility in any variety thereby paving the way for the dissemination of all possible hybrids using seeds, without being limited by the rarity of naturally occurring sterile males.
